# Small RNA and PARE sequencing in flower bud reveal the involvement of sRNAs in endodormancy release of Japanese pear (*Pyrus pyrifolia* 'Kosui')

**DOI:** 10.1186/s12864-016-2514-8

**Published:** 2016-03-15

**Authors:** Songling Bai, Takanori Saito, Akiko Ito, Pham Anh Tuan, Ying Xu, Yuanwen Teng, Takaya Moriguchi

**Affiliations:** NARO Institute of Fruit Tree Science, Tsukuba, Ibaraki 305-8605 Japan; Department of Horticulture, The State Agricultural Ministry’s Key Laboratory of Horticultural Plant Growth, Development & Quality Improvement, Zhejiang University, Hangzhou, Zhejiang Province 310058 China; Present address: Graduate School of Horticulture, Chiba University, Matsudo-shi, Chiba 271-8510 Japan

**Keywords:** Endodormancy, Flower bud, Japanese pear, PARE-seq, Small RNA, sRNA-seq

## Abstract

**Background:**

In woody perennial plants, including deciduous fruit trees, such as pear, endodormancy is a strategy for surviving the cold winter. A better understanding of the mechanism underlying the endodormancy phase transition is necessary for developing countermeasures against the effects of global warming. In this study, we analyzed the sRNAome of Japanese pear flower buds in endodormant and ecodormant stages over two seasons by implementing of RNA-seq and degradome-sequencing.

**Results:**

We identified 137 conserved or less conserved miRNAs and 50 pear-specific miRNAs. However, none of the conserved microRNAs or pear-specific miRNAs was differentially expressed between endodormancy and ecodormancy stages. On the contrast, 1540 of 218,050 loci that produced sRNAs were differentially expressed between endodormancy and ecodormancy, suggesting their potential roles on the phase transition from endodormancy to ecodomancy. We also characterized a multifunctional miRNA precursor *MIR168,* which produces two functional miR168 transcripts, namely miR168.1 and miR168.2; cleavage events were predominantly mediated by the non-conserved variant miR168.2 rather than the conserved variant miR168.1. Finally, we showed that a *TAS3* trans-acting siRNA triggered phased siRNA within the ORF of one of its target genes, *AUXIN RESPONSE FACTOR 4*, via the analysis of phased siRNA loci, indicating that siRNAs are able to trigger phased siRNAs in pear.

**Conclusion:**

We analyzed the sRNAome of pear flower bud during dormant phase transition. Our work described the sRNA profiles of pear winter buds during dormant phase transition, showing that dormancy release is a highly coordinated physiological process involving the regulation of sRNAs.

**Electronic supplementary material:**

The online version of this article (doi:10.1186/s12864-016-2514-8) contains supplementary material, which is available to authorized users.

## Background

Small RNAs (sRNAs) are ubiquitous regulatory molelcules produced by many thousands of endogenous genes. Silencing pathways based on sRNAs that function at the transcriptional or post-transcriptional levels to negatively regulate the expression of protein-encoding genes have been identified in most eukaryotes. These pathways are also considered to play a role in the silencing of viral RNA and the suppression of mobile elements [1, 2]. Plant sRNAs can be categorized into several major classes, including microRNA (miRNA), heterochromatic small interfering RNA (hc-siRNA), and phased/secondary siRNA (pha-siRNA), according to their origin and biosynthesis [3, 4]. miRNAs are transcribed from miRNA (*MIR*) gene families, which occur mainly in the intergenic genomic region and sometimes inside protein-coding genes; transcripts eventually become mature miRNAs (mainly 20–22 nt long) via processing by DICER-LIKE1 (DCL1) protein [5]. siRNA is processed from double stranded RNA (dsRNA) or from long, perfectly complementary hairpin RNA molecules. dsRNA is recognized and cleaved by DCL2/3/4 to generate different classes of siRNA. hc-siRNA is 24 nt long and results from DCL3 processing of dsRNA transcribed from intergenic or repetitive regions of the genome by the plant-specific RNA polymerase Pol IV, and possibly also Pol V [6–8]. hc-siRNA functions to maintain genome integrity by suppressing transposable elements via an RNA-dependent DNA methylation (RdDM) pathway [4]. In contrast, pha-siRNA is processed by DCL4 from dsRNA dependent on RNA-dependent RNA polymerase 6 (RDR6). A well-described category of pha-siRNA includes trans-acting siRNA (ta-siRNA) of *Arabidopsis* [9]. While hc-siRNA plays a crucial role in chromatin modification, miRNA and pha-siRNA function mainly at the posttranscriptional level, via either cleavage or translational suppression of target transcripts, and in a few instances also directing the methylation of DNA [10, 11].

miRNA has been implicated in the control of diverse cellular, physiological and developmental processes in plants. Among plant species, there are several miRNA species with well-conserved sequences and functions. For example, miR156 targets a series of *SQUAMOSA-PROMOTER BINDING PROTEIN-LIKE* (*SPL*) genes, regulating the phase transition from juvenile to adult during shoot development [12]. miR172 acts downstream of miR156 and mediates regulation of *APETALA2* (*AP2*) and *AP2*-like genes, which are needed for proper specification of flower organs [13]. The relative balance of miR156 and miR172 is essential for the regulation of phase change and flowering [14]. miR390 directs cleavage of *trans-acting siRNA 3* gene (*TAS3*), leading to production of ta-siRNAs [9], which, together with miR160 and miR167, targets the *AUXIN RESPONSE FACTOR* (*ARF*) gene family, regulating the response of auxin in plant cells [15, 16].

Recent bioinformatics and high-throughput sequencing studies have uncovered a large number of non-conserved miRNAs. These are mostly expressed at low levels with divergent target genes; thereby, they may have specialized functions. Several non-conserved miRNAs indeed function in fine-tuning roles in the target regulatory networks of different plants [17–20]. Discoveries of specific mechanisms and functions of non-conserved plant miRNAs over a wide range of conditions are an expanding topic of investigation [21]. In pome fruits, some progresses in the identification of miRNAs have been achieved. Xia et al. [22] identified apple miRNAs and pha-siRNAs, describing novel regulatory networks targeting a multitude of genes inside and outside the MYB family. Visser et al. [23] further extended the apple sRNAome by characterizing miRNAs and siRNAs in apple leaves. Ma et al. [24] suggested that an apple-specific miRNA may affect the disease resistance pathway by targeting a group of *NUCLEOTIDE BINDING SITE-LEUCINE-RICH REPEATS* (*NBS-LRR*) resistance genes. In addition, Niu et al. [25] *in silico* predicted miRNAs using the genome sequence of Chinese pear (*Pyrus pyrifolia* ‘white pear’ group), providing a basic resource to support future research in this specie.

In woody perennial plants, including deciduous fruit trees, such as pear, endodormancy is a strategy for surviving the cold winter. To complete endodormancy and resume growth, low temperatures are required, but the recent global warming trend has sometimes interrupted normal endodormancy establishment and release [26]. Therefore, elucidation of the mechanism underlying the endodormancy phase transition is necessary for developing countermeasures against the effects of global warming. In a previous study, we carried out transcriptome analysis of Japanese pear flower buds during endodormancy phase transition and demonstrated changes in transcript abundance for genes involved in phytohormone metabolism, antioxidant response and methylation changes [27]. We also focused on the function of *dormancy-associated MADS-box* genes. However, whether sRNAs were involved in the phase transition during dormancy has thus far not been well examined.

In this study, we analyze the sRNAome of Japanese pear flower buds in endodormant and ecodormant stages over two seasons through next-generation sequencing. By implementing of RNA-seq and parallel analysis of RNA end sequencing (PARE-seq), we found that several sRNA loci differential expressed between endodormancy and ecodormancy stages, demonstrating possible involvement in the regulation of endodormancy release. Moreover, we observed a multifunctional miRNA precursor *MIR168* and the pha-siRNA production within the ORF of *ARF4* triggered by a ta-siRNA. This work constitutes the study to characterize sRNAs in pear winter buds and provides a platform for further investigation of specific sRNAs in various biological processes in pear.

## Results

### High-throughput sequencing of sRNAs in winter flower buds

The plant samples used in the present study have been described in previous reports [27]. sRNA libraries were constructed from flower buds collected on December 6 (endodormancy) and December 31 (ecodormancy) of the 2009/2010 winter season and on December 2 (endodormancy) and December 31 (ecodormancy) of the 2010/2011 season. As endodormancy released in the late December in both years according to the DVI model [27, 28], our samples thus designated as endo2009, eco2009, endo2010 and eco2010 for respective buds from December 6 and 31 of the 2009/2010 season and from December 2 and 31 of the 2010/2011 season [27]. High-throughput sequencing generated at least eighteen million clean reads per sample (Table 1). The total numbers of reads are similar in all the samples, allowing a meaningful comparison between endodormant and ecodormant stages. After collapsing into unique reads, 5.2–7.7 million unique sequences were obtained in each library.

**Table 1:** Summary of Next-generation sequencing of small RNAs

	Endo2009		Endo2010		Eco2009		Eco2010	
	Redundant reads	Unique reads	Redundant reads	Unique reads	Redundant reads	Unique reads	Redundant reads	Unique reads
Clean reads	27873255	7691122	23409758	7572632	23790014	6744685	18048756	5220127
Repeat-associated sRNA	6435475	741816	4605822	736636	5248276	642142	4460758	513072
rRNA	2285572	70600	1394647	68056	1747779	68694	1646423	74493
snRNA	17635	3639	12288	3308	15218	3456	12566	3145
snoRNA	5696	1458	2843	1040	4361	1295	3302	1038
tRNA	2944036	14176	1663477	13764	2752460	14053	1966155	16495
sRNAs (between 18 and 25 nt) with reads > =2	16182369	2043076	14254330	1959093	13505620	1766538	9171710	1248574

**Fig. 1:**
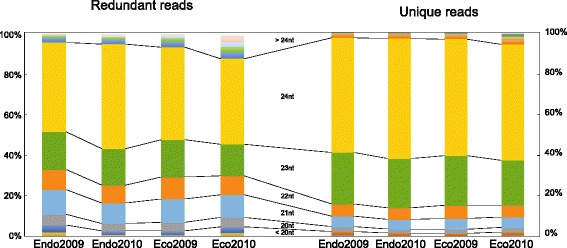
Size distribution of redundant reads and unique reads in the four libraries used in this work. Samples “Endo2009” and “Endo2010” were collected on Dec. 6, 2009 and Dec. 2, 2010 (under endodormancy), respectively. Samples “Eco2009” and “Eco2010” were harvested and on Dec. 31, 2009 and Dec. 31, 2010 (after endodormancy release = ecodormancy), respectively

Reads of 19–25 nt accounted for over 95 % of the total reads, among which about half were 24 nt long in all four libraries (44.2–51.9 %, Fig. 1). The second most abundant size was 23 nt (~20 %), followed by 21 nt (~10 %) and 22 nt (~10 %). The sRNA sequences were then annotated with repeat-associated sequences and the non-coding RNA database (Rfam 10.0) [29]. Sequence reads from rRNA, tRNA and repeat regions made up about 10 % of the unique sequences and 20 % of the redundant sequences in all four samples (Table 1). For further analysis, reads of length 19-25 nt that were sequenced at least twice were mapped to the Chinese pear genome (Pbr1.0, downloaded from http://peargenome.njau.edu.cn; [30]). About 80 % of the sequences mapped to at least one scaffold and were further analyzed.

### De novo annotation of sRNA loci in the pear genome

**Fig. 2:**
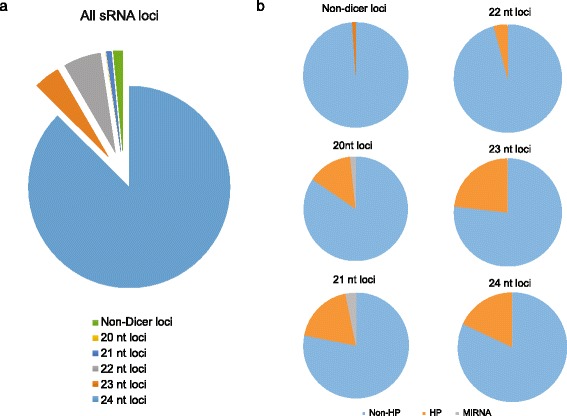
sRNA clusters (loci) identified by ShortStack pipeline. In total, 218,050 clusters were identified. **a** distribution of sRNA loci generating different sRNA species. 21 nt loci were processed by DCL1/4, 22 nt loci were processed by DCL2, and 23, 24 nt loci were processed by DCL3. Note that most sRNA loci in pear generated 24 nt sRNAs. **b** distribution of sRNA loci with different sequence type. MIRNA loci produce miRNAs, HP loci and non-HP loci produce 21–24 nt siRNAs

After filtering the reads derived from rRNA and tRNA, clean reads were aligned to the pear genome using Bowtie (v1.0.0, [31]) and were analyzed with ShortStack [32] using the default “plant mode.” In total, 218,050 loci were identified as producing sRNA (Additional file 1: Table S1), among which 164 miRNA loci (*MIR* genes) and 37,212 other hairpin (HP) RNA loci were identified. The sRNA derived from most of these loci is putatively processed by dicers, but 3218 loci were annotated as non-dicer processed. The sRNA loci were grouped according to mapped sRNA length. As shown in Fig. 2, 24 nt loci were the most abundant (87.4 %), while 20-21 nt loci occurred least frequently. In all the loci groups, non-HP loci accounted for over 75 % of all loci. In addition, most of the miRNA loci were categorized as 21 or 22 nt.

### Summary of PARE-seq

**Fig. 3:**
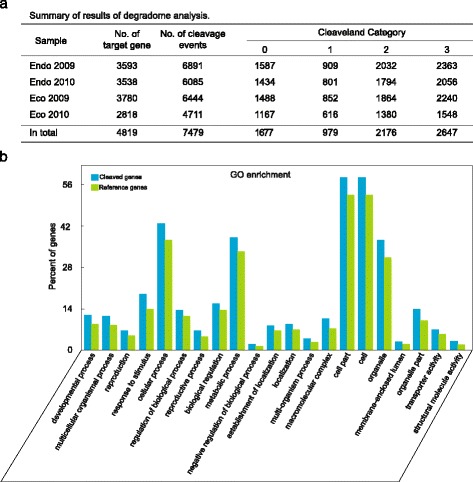
PARE-seq to identify the sRNA-mediated cleavage in pear winter buds. The mixed samples used for sRNA-seq were also used for PARE-seq. **a** sRNA-mediated transcript cleavage identified with the PAREsnip pipeline. The sRNAs with 20–22 nt, ≥10 reads in at least one sample were used for analysis. Category 4 was not included to avoid false positive results. **b** GO enrichment analysis of cleaved genes identified in (**a**). Only significantly enriched GO categories are shown

PARE-seq of winter flower buds produced 16,986,045 clean reads with sizes of 20 and 21 nt, corresponding to 4,531,717 non-redundant tags, among which 2,055,412 tags were successfully mapped to 32,944 pear transcripts [30]. To describe transcriptome-wide cleavage events, we performed the analysis using the well-established pipeline PAREsnip [33]. In total, 7477 cleavage events (Additional file 2: Table S2) were found in 4819 genes with statistical significance. These cleavage events fell into categories 0 to 3 (category 0 represented the cleavage position with the maximum depth (>1 read) on the transcript and there is just 1 position at the maximum value on the transcript; category 1 represented the position with the depth equal to the maximum value (>1 read) on the transcript, and there is >1 position at maximum value; category 2 represented the position with the depth (>1 read) above the average depth, but not the maximum on the transcript; category 3 represented the position with the depth (>1 read) below or equal to the average depth of coverage on the transcript) defined in PAREsnip (to avoid false positive results, we did not use category 4, which represented cleavage events supported by only one read mapped). As shown in Fig. 3a, 22.4 % of cleavage events belonged to category 0, which was defined as the most abundant cleavage event on a transcript. The cleaved genes were then subjected to GO enrichment analysis. The results showed that “responds to stimulus” was enriched with the lowest false discovery rate (1.2e-09), suggesting a role for miRNA in regulation of responses to biotic and/or abiotic stimuli. In addition, metabolic process and other 19 categories were significantly enriched (Fig. 3b).

### Identification of known miRNAs in pear winter flower buds

*MIR* loci identified by ShortStack were compared to digitally predicted miRNAs [25]. Of 164 annotated *MIR* loci, 47 overlapped with previously predicted *MIR* loci, which generated 68 mature miRNAs (including both the 5p and 3p miRNAs from the same precursor). These *MIR* loci produce miRNAs in 22 miRNA families, including 15 conserved miRNAs and seven less-conserved miRNAs (data not shown).

**Fig. 4:**
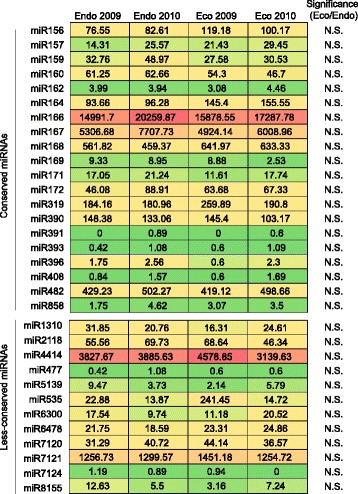
Identification and differential expression analysis of known miRNAs in pear winter buds between endodormancy and ecodormancy. Normalized read counts (RPM) of conserved and less-conserved miRNAs in the four samples are shown

As the relatively high stringent ShortStack algorithm tended to produce false negative results [32], BLAST-based strategies were also used to identify known miRNAs with miRProf [34]. The clean reads were compared to a publicly available miRNA database [35]. When only allowing perfect matches, a total of 137 unique sequences were annotated as known miRNAs in the four sRNA libraries. They clustered into 32 miRNA families, including 20 conserved families and 12 less-conserved families (Fig. 4). The abundance of individual conserved miRNAs ranges from several reads to a few million reads in the libraries. These miRNAs showed similar expression patterns in the four libraries: miR166 was the largest represented miRNA family, followed by miR167, both of which have over 1000 normalized reads in all four libraries. Six miRNAs (miR156, miR164, miR168, miR319, miR390 and miR482) have normalized read counts between 100 and 1000 in at least one library. Compared to apple, which contains 206 mature miRNAs belonging to 43 families, the pear winter buds lacked several sRNA families, such as miR828, probably due to their temporal- or spatial- specific expression that was also observed in apple [22].

Targets of miRNAs were identified using PARE-seq data. A total 131 genes were identified as targets of conserved miRNAs. Representative targets of conserved miRNAs are shown in Additional file 3: Table S3. As previously reported, most conserved miRNAs targeted gene families. Although most (122) of the 131 identified genes were conserved targets for these miRNAs across a wide range of plant species, nine had not been reported in other species. For example, miR166, which is known to target homeobox proteins in plant, was found to target genes encoding fructose-bispospate aldolase and glutathione peroxidase. Similarly, miR167, which targeted a series of *ARF* genes, also targeted a gene similar to the *Arabidopsis RESPONSIVE TO DEHYDRATION 19* (*RD19*) gene. About two-third of the cleavage events (85 of 131) mediated by conserved miRNAs were in category 0, with the maximum depth on the transcripts.

### Identification of pear-specific miRNAs and their targets

To identify pear-specific miRNAs, we used two well-established pipelines, ShortStack and miRCAT [34]. As mentioned above, ShortStack identified 164 miRNA loci, among which 47 were annotated as known miRNAs. The remaining 117 *MIR* loci were considered specific to pear. Among these 117 loci, 12 loci produced mature miRNAs of 21–22 nt (Table 2), while others generated 23–24 nt miRNAs. Meanwhile, after removing known miRNAs, the remaining 20–22 nt sRNA reads were subjected to the miRCAT pipeline using default parameters for plant miRNA identification. A total of 50 miRNA candidates were identified from the four libraries, of which 7 had miRNA* (star strand) sequences identified from the same libraries, while the other 43 had no identified miRNA* sequences (Table 2). In total, we identified 16 candidates with miRNA* (3 candidates were identified by both miRCAT and ShortStack), which were considered as pear-specific miRNAs, and 43 candidates without miRNA* sequences were considered as pear-specific miRNA candidates. Stem-loop structures of the predicted precursors of each pear specific miRNAs were shown in Additional file 3: Figure S1.

**Table 2:** Novel or candidate miRNAs found in pear bud^a^

Name	miRNA _sequence	Length	Contig	miRNA* sequence	Pipeline used^b^
miRn1	TGCGTTTGCACCTGCACCTCT	21	scaffold303.0	AAGGTGCAGGTGTAACTGCAG	miRCAT
miRn2	CGAAGACCTTGGGGAGAGTGG	21	scaffold1160.0	ACTCTCCCTCAAGGGCTTCGA	miRCAT
miRn3	AAGCGGAGTAATGGTTACTGA	21	scaffold276.0	AGAGCTGCATTATTCTCTTGT	ShortStack
miRn4	TCACCTTGTAAAAAATTGGCC	21	scaffold266.0	CAATTTTTTATAAGGTGAAT	ShortStack
miRn5	TCGGGACAGCGTACTTGAGTT	21	scaffold230.0	CTCACGCGCGCTGTCCCGAGA	ShortStack
miRn6	TTGAGGATGCATAGTTTTCAG	21	scaffold266.0	GAAAATAATGCATCTTCAACG	ShortStack, miRCAT
miRn7	GTGTAACCGTCGTAATGTCCC	21	scaffold198.0	GACATTACAACGGCCACACGG	ShortStack
miRn8	CTGCCAAAGGAGATCTGCTCAG	22	scaffold1.0	GAGCAGTCTCCTCTTGGCAAAC	ShortStack,miRCAT
miRn9	TCCCTAAAACCACCAAGGCCAA	22	scaffold2.0	GGCTTTAGTGGGTTAGGAAGA	ShortStack
miRn10	GCGTACGAGGAGCCAAGCATA	21	scaffold32.0	TGCCTGGCTCCCTGTATGCCA	miRCAT
miRn11	AATAATTGTAGTCGTTGGATCA	22	scaffold371.0	TTTGATCTAACGGCTACAAACA	miRCAT
miRn12	TGGTGTTTGGATGGACGTGTT	21	scaffold862.0	TAAGTTCATCCAAACACCATA	ShortStack
miRn13	AGGTGCAGGTGTAACTGCAGA	21	scaffold303.0	TGCGTTTGCACCTGCACCTCT	ShortStack
miRn14	ACCTAGCTCTGATACCATAAA	21	scaffold3.0	TGTGGTATCAGGACTATGTTA	ShortStack
miRn15	GGTTTGAGTGGATTGGGAAGA	21	scaffold2.0	TTCTTAAAACCACTCAAGCCAA	ShortStack, miRCAT
miRn16	TTCTTGACCACCGACGCCGACA	22	scaffold131.0	TTGGCGTCGTGGTCAAGAAGG	ShortStack
miRn17	AAAACCGCGGATTGGGGCGTG	21	scaffold17.0	NO	miRCAT
miRn18	AAGAGATATGGACCGTTGGATA	22	scaffold1.0	NO	miRCAT
miRn19	AATGACGTGTGGCATATCATC	21	scaffold28.0	NO	miRCAT
miRn20	ACACGATGTATGATGAACGG	20	scaffold1044.0	NO	miRCAT
miRn21	ACCTGATTGGTTGCTGTTGGAT	22	scaffold89.0	NO	miRCAT
miRn22	ACTTTGGGATGTGGCAATGTGA	22	scaffold285.0	NO	miRCAT
miRn23	AGGAGACGAAGAAACTGGTGC	21	scaffold463.0	NO	miRCAT
miRn24	AGGAGAGGGAGGTGGGTCGGG	21	scaffold6.0	NO	miRCAT
miRn25	AGGATGAGCTGAAGATGATAA	21	scaffold1.0	NO	miRCAT
miRn26	CAAAGTTTTTGGAATGTTGCA	21	scaffold54.2	NO	miRCAT
miRn27	CCGAACTTGGTGGATTAGGAG	21	scaffold1.0	NO	miRCAT
miRn28	CCGGATTTCGTGGTCAGGAGG	21	scaffold124.0	NO	miRCAT
miRn29	CCTGACTGTTGATGCATGTAGG	22	scaffold373.0	NO	miRCAT
miRn30	CCTTGTTTAGGGTATGTAGGCA	22	scaffold1113.0	NO	miRCAT
miRn31	CGAACTTGGTGGATTAGGAGG	21	scaffold30.0	NO	miRCAT
miRn32	CGAGGGTGTGGTGTAGGGTGG	21	scaffold181.0	NO	miRCAT
miRn33	CGATGAACGGACGTGATTGGA	21	scaffold10.0	NO	miRCAT
miRn34	CGATGTACGATGAACGGACACA	22	scaffold1279.0	NO	miRCAT
miRn35	CGCCTTGGCGAAACTCTAGGAA	22	scaffold310.0	NO	miRCAT
miRn36	CGGAATACGGATGGTACACCA	21	scaffold63.0	NO	miRCAT
miRn37	CGGATTTCGTGGTCAGGAGGA	21	scaffold228.0	NO	miRCAT
miRn38	CGGTCAGTAGGATCCCAAGGCA	22	scaffold242.0	NO	miRCAT
miRn39	CGTACGATATACAATGAACGGT	22	scaffold171.0	NO	miRCAT
miRn40	CGTACGATCTACGATGAACGG	21	scaffold182.0	NO	miRCAT
miRn41	CTAATGACATTATAGAGGACA	21	scaffold103.0	NO	miRCAT
miRn42	CTGCACAATGTACGATGAACGG	22	scaffold13.0	NO	miRCAT
miRn43	CTGTACGATATATGATGAACGG	22	scaffold1196.0	NO	miRCAT
miRn44	GAATACGGATGGTACACCAT	20	scaffold330.0	NO	miRCAT
miRn45	GCATTTGGATTTGTCTGACTTG	22	scaffold1206.0	NO	miRCAT
miRn46	GGGACTTTCAAATTCCGAGGG	21	scaffold353.0	NO	miRCAT
miRn47	GTCAAACTGTGATTTGTAGGCA	22	scaffold94.0	NO	miRCAT
miRn48	GTGACTGATGACATGTTGTAG	21	scaffold322.0	NO	miRCAT
miRn49	TAGGCGTAGATAGACCGTGGG	21	scaffold144.0	NO	miRCAT
miRn50	TCAAATTTCAAAGGTCCGGATC	22	scaffold221.0	NO	miRCAT
miRn51	TCACACTATGGAGCGATGGTC	21	scaffold1.0	NO	miRCAT
miRn52	TCCTCGAACTGTAGCAATGGC	21	scaffold44.1	NO	miRCAT
miRn53	TCTAACGGTCAAGAGGATGTC	21	scaffold751.0	NO	miRCAT
miRn54	TCTGGATGCATGAATTTGGTA	21	scaffold83.0	NO	miRCAT
miRn55	TGAACGGACATGATTTGAGGA	21	scaffold3.0	NO	miRCAT
miRn56	TGGATTTGGTAGAAGGGATCC	21	scaffold12.0	NO	miRCAT
miRn57	TGTGATGTGTGGTTACGGTT	20	scaffold235.0	NO	miRCAT
miRn58	TTCAGGGCTCTGAGTGGGATGG	22	scaffold48.0	NO	miRCAT
miRn59	TTGGATTAAAATTGAACGGCC	21	scaffold114.0	NO	miRCAT

^a^Additional file 4: Figure S1 showed the stem-loop structure of the predicted precursors of each pear-specific miRNAs

^b^Pipeline(s) that successfully identified the miRNA loci

Of the 59 pear-specific miRNAs, 37 belonged to the 21 nt class of miRNAs and 19 belonged to the 22 nt class, while the remaining three were 20 nt long (Table 2). In general, the pear-specific miRNAs were much less abundant than the conserved miRNAs in our libraries. For example, in the endo2009 dataset, only 4 of the 59 novel miRNAs yielded levels over 10 RPM, while 37 were below 1 RPM (Additional file 5: Table S4).

Target genes were also identified for the 59 pear-specific miRNAs. Twenty-nine genes were identified as targets of 16 pear-specific miRNAs (Table 3). Of the 29 target genes, two belonged to category 0, three to category 1 and eight to category 2, while all others were classified into category 3. Among these 29 genes, eight genes were successfully annotated by TAIR 10. The target of miRn20 encoded *GAMMA CARBONIC ANHYDRASE-LIKE 2*, while miRn35 targeted a *NITRATE TRANSPORTER* gene. In addition, miRn42 targeted *POLYUBIQUITIN 10* (*UBQ10*) and miRn52 targeted an *ACTIN-RELATED PROTEIN 5* (*ARP5*). Hence, these pear specific miRNAs may be involved in the regulation of an array of metabolic and biological processes and signaling pathways. Similar to previous reports, however, most of the pear-specific miRNA targets fell into categories 2 or 3, which represents a relatively low-confidence group and necessitates further experimental validation.

**Table 3:** Target of pear-specific miRNAs in pear identified by degradome sequencing

ID	Gene	Cleavage Position	Category	Allan Score	*P*-Value	TAIR annotation	Gene description
miRn1	Pbr011776.1	240	2	2.5	0.05	AT5G43630	TZP
miRn1	Pbr018332.1	240	2	2.5	0.01	AT5G43630	TZP
miRn4	Pbr011743.1	328	0	2	0	AT1G56510	ACTIVATED DISEASE RESISTANCE 2
miRn6	Pbr011682.2	253	2	1.5	0	AT5G36930	NA
miRn6	Pbr030895.1	271	2	2	0.01	AT5G17680	NA
miRn8	Pbr039988.1	1450	2	3	0.05	AT1G54130	RELA/SPOT HOMOLOG 3
miRn9	Pbr038135.1	1057	2	3.5	0.02	AT4G30110	heavy metal atpase 2
miRn11	Pbr000099.1	885	2	3.5	0.05	AT1G12000	NA
miRn13	Pbr027465.1	111	0	4.5	0.02	AT3G25800	PR 65
miRn18	Pbr002749.2	2267	3	4	0.05	AT4G10320	NA
miRn23	Pbr009493.1	78	2	2.5	0	AT2G29670	NA
miRn24	Pbr026566.1	243	2	2	0.03	AT4G24330	NA
miRn24	Pbr004924.1	978	0	4	0.02	AT3G52110	NA
miRn31	Pbr036178.1	256	3	3.5	0.04	AT3G26510	NA
miRn32	Pbr006676.1	1157	0	4	0.02	AT5G26710	NA
miRn38	Pbr029169.1	420	3	4	0.04	AT4G02040	NA
miRn38	Pbr007316.1	420	3	4	0.04	AT4G02040	NA
miRn41	Pbr034171.1	167	3	3.5	0.02	AT2G35680	NA
miRn41	Pbr027934.1	1315	2	3	0.05	AT5G64030	NA
miRn41	Pbr024612.1	167	3	3.5	0.03	AT2G35680	NA
miRn41	Pbr013804.1	1105	3	3.5	0.01	AT2G02955	maternal effect embryo arrest 12
miRn41	Pbr030334.1	899	0	4	0.02	AT5G60770	nitrate transporter 2.4
miRn41	Pbr024607.1	167	3	3.5	0.01	AT2G35680	NA
miRn41	Pbr029396.1	167	3	3.5	0.03	AT2G35680	NA
miRn46	Pbr035452.1	735	3	3	0.02	AT4G05320	polyubiquitin 10
miRn54	Pbr000659.1	966	2	2.5	0.02	AT5G13030	NA
miRn54	Pbr005201.1	1526	1	4	0	AT5G35690	NA
miRn55	Pbr009389.1	551	1	4.5	0.04	AT3G12380	actin-related protein 5
miRn57	Pbr039571.2	962	2	2.5	0.03	AT2G34470	urease accessory protein G

### Differential expression of sRNAs during endodormancy release

Differential expression analysis of sRNAs was performed on two levels: individual sequences of miRNAs and clusters annotated by ShortStack. Differential expression was defined as at least a 2-fold (upregulation) or 0.5-fold (downregulation) change, with statistically significant difference between ecodormancy and endodormancy samples.

**Fig. 5:**
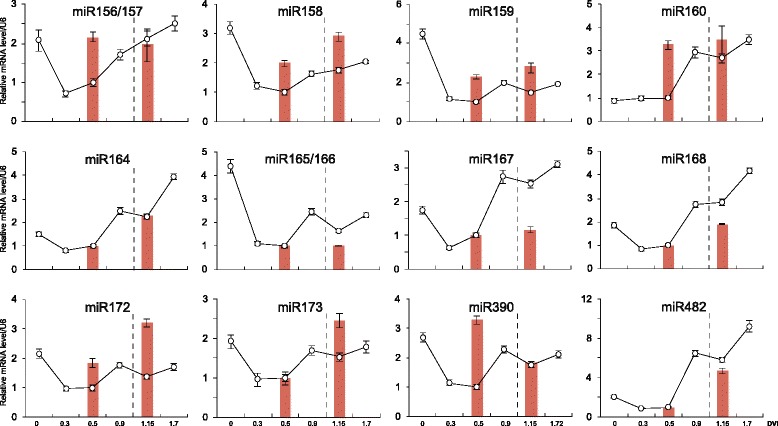
qRT-PCR validation of the expression of conserved miRNAs. Samples of 6 time points were used for analysis in the season of 2009/2010 (*line*) and only samples of 2 time points were analyzed in the season of 2010/2011 (*bars*) due to the lack of enough samples. Error bars indicate the standard deviation of three technical replicates. The date corresponding to endodormancy-release was marked with dash line

The differential expression analysis between endodormancy and ecodormancy stages of miRNAs was performed using edgeR [36]. Two-year samples of similar dormancy stages, judged by DVI values, were used as biological replicates. In our datasets, we did not observe any differentially expressive conserved miRNA (Fig. 4). Furhtermore, we carried out qRT-PCR to confirm their expression patterns, and most of the miRNA expression patterns were similar as that determined by the next-generation sequencing (Fig. 5). In our work, the samples collected from two successive seasons allowed us to identify whether the miRNAs were stably differential expressive through different years. As shown in Fig. 5, although some miRNAs, e.g. miR164, miR167, significantly differential expressed between DVI 0.5 and DVI 1.15 in the 2009/2010 season, their expression patterns in the samples within the similar dormant stages in 2010/2011 season were totally different, suggesting that such differential expressions were not related to the dormant stage transition but to other reasons such as environmental changes and so on. In addition, the pear-specific miRNAs were also analyzed, but none of them showed differential expression between endodormancy and ecodormancy buds (data not shown).

**Fig. 6:**
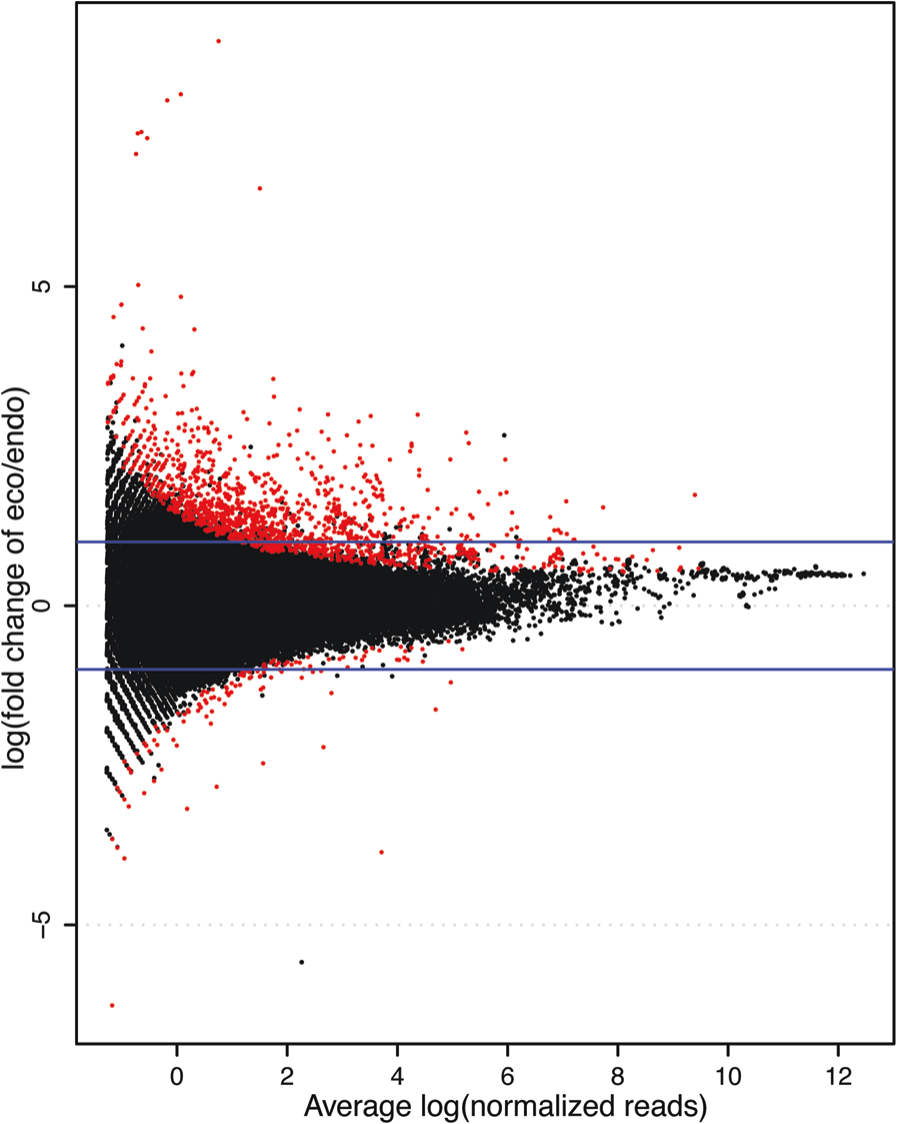
MA-plot of differential expression analysis of sRNA loci between endodormancy and ecodormancy. Note that the up-regulated sRNA loci were much more that the down-regulated loci

**Fig. 7:**
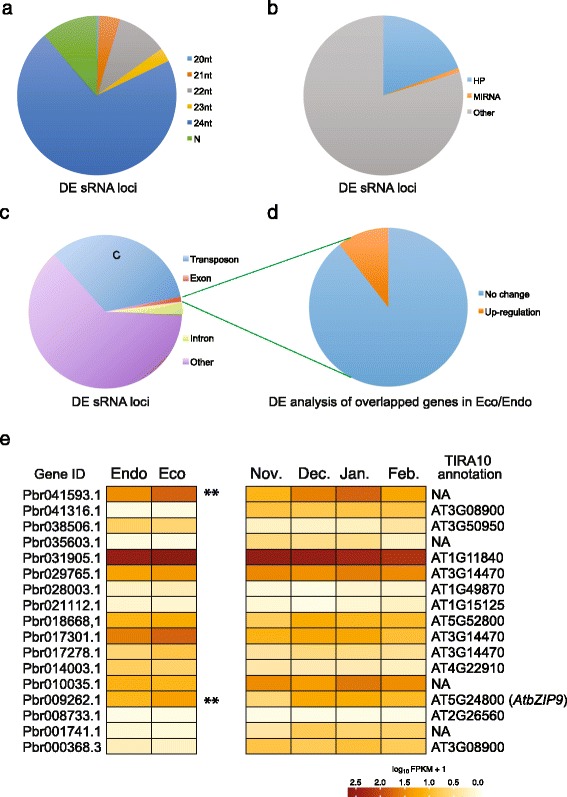
Annotation of differential expressed sRNA loci identified the putative cleavage of transcripts in pear winter buds. **a** Distribution among length groups of DE sRNA loci. **b** Distribution among structure groups of DE sRNA loci. **c** A small portion of sRNA loci overlapped with transposon and transcripts. **d** Especially, 20 loci located in exon regions of 19 genes, within which two genes were upregulated in pear winter buds toward endodormancy release. **e** Expression pattern of the DE-sRNA-loci-overlapped genes in ‘Kosui’ and ‘Suli’. Two genes, a *bZIP* gene and an unannotatable gene, were differentially expressed between endodormant and ecodormant buds

Differential expression analysis of sRNA loci was also performed with edgeR. Among the 218,050 sRNA loci, a total of 1540 differential expression loci were identified, of which 1287 were upregulated and 253 were downregulated in the ecodormancy stage (Fig. 6). The imbalance between up- and down-regulated loci might suggest increased production of sRNAs in ecodormancy pear winter buds. As shown in Fig. 7a, ~70 % of the differentially expressed loci belonged to the 23–24 nt group, while 30 % were categorized as 20–22 nt. In terms of structure, 14 differentially expressed loci were *MIR* loci and 299 were HP loci, while the rest were non-HP loci (Fig. 7b). Among these differential expressive loci, 571 loci were annotated as transposons and 66 loci overlapped with annotated transcripts, including 46 located in introns and 20 in exons (Fig. 7c). All 20 exon-overlapped loci were upregulated at ecodormancy. As the presence of exon-originated siRNA suggested DCL-mediated cleavage of transcripts, the corresponding transcript abundances were analyzed using RNA-seq data (Fig. 7d and e). As a result, the 20 exon-overlapped loci were found located in 19 genes, two of which showed significant upregulation at ecodormancy (Fig. 7e): Pbr009262.1, similar to *AtbZIP9* (AT5G24800) and an un-annotatable gene Pbr041593.1. The remaining genes were stably expressed near endodormancy release in both ‘Kosui’ and ‘Suli’ libraries (Fig. 7d and e).

### Identification of the multi-functional precursor of miR168

**Fig. 8:**
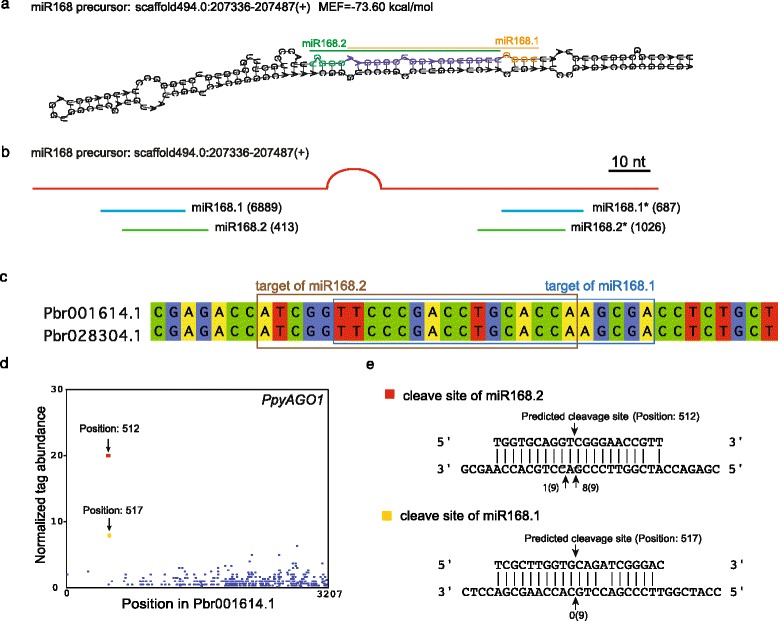
Identification of a multi-functional miRNA precursor of miR168 in pear winter buds. **a** The secondary structure of the miR168 precursor. The regions producing miR168.1 and miR168.2 are indicated. **b** Abundances of miR168.1, miR168.2 and their star sequences produced from the precursor. Endo2009 was used as the example. **c** The binding sites of miR168.1 and miR168.2 on two target transcripts. **d** T-plot indicated the cleavage events mediated by miR168.1 and miR168.2 based on the PARE-seq. **e** Cleavage sites mediated by miR168.1 and miR168.2. The RML-RACE were applied to confirm the cleavage events identified with PARE-seq

It has been shown that miR168 is involved in the post-transcriptional regulation of *AGO1* in *Arabidopsis* and other plant species [37]. In pear winter flower buds, we observed a similar cleavage event via PARE-seq. However, this cleavage event was in category 2, while second cleavage event, located 5 bp upstream and in category 0, was mediated by an unknown 21 nt sRNA with two variants, between which there was a SNP near the 3′ end. Alignment with the known miR168 sequence showed that the 16 nt sequence at the 5′ end of the unknown sRNAs perfectly matched 16 nt at the 3′ end of miR168. Analysis of their precursors showed that the unknown sRNAs and miR168 derived from overlapping position within the same precursor, while differing in abundance (Fig. 8a and b). These results suggested that in pear, the miR168 precursor was able to produce multiple functional mature miRNAs. We thus named the two miRNAs miR168.1 and miR168.2. However, miR168.1 was the major miR168 species in all four libraries, e.g., in the endo2009 library, miR168.1 and its miRNA* account for over 80 % of sRNA produced from the precursor, while miR168.2 accounts for only ~5 % (Fig. 8b). We found, though, that miR168.2 dominantly mediated *AGO1* cleavage in pear winter buds (Fig. 8c and d). This was further confirmed by 5′-RACE (Fig. 8e); in all nine independently sequenced colonies, the 5′ end corresponded to the cleavage site of position 512, which was mediated by miR168.2.

### Identification of TAS3 locus and the pha-siRNA locus triggered by TAS3 ta-siRNA

**Fig. 9:**
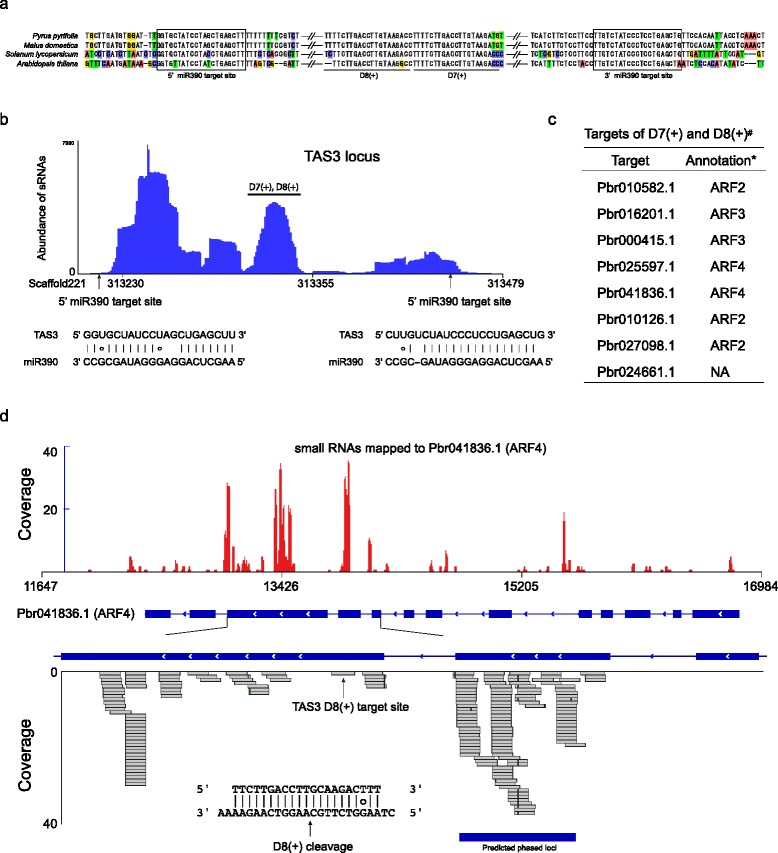
Identification of *TAS3* locus and the target of *TAS3*-siRNAs. **a** Comparison of pear *TAS3* locus with that of other plant species. **b** *TAS3*-siRNAs produced from the *TAS3* locus. The dual target sites of miR390 and the region producing conserved ta-siRNAs were indicated. **c** PARE-seq identified the targets of the conserved *TAS3*-siRNAs D7(+) and D8(+). **d** D7/8(+) triggered the production of phased siRNAs upstream of the target site in Pbr041836.1 (*ARF4*)

In the present study, identification of pha-siRNA was performed using ShortStack, by implementing the pear genome sequences. In total, 133 phased clusters were predicted to be statistically significant. For further analysis, 32 loci with hairpin structure and 18 loci annotated as miRNA precursors were excluded. Among the remaining loci, we identified a *TAS3* locus (Fig. 9a) with two miR390 target sites with Allan scores of 4.5 and 5.5 (Fig. 9b). Alignment of the *TAS3* locus with those of other plant species revealed conservation of the two target sites as well as a region for the production of *ARF*-targeting ta-siRNAs (Fig. 9a and b). The conserved *TAS3*-derived pha-siRNAs D7(+) and D8(+) and the PARE data were subsequently submitted to the CleaveLand pipeline. Seven *ARF* genes, and an unannotated gene (Pbr024661.1), were identified as targets of D7(+) and D8(+) (Fig. 9c). Interestingly, the upstream region of the D8(+) target site in Pbr041836.1 (*ARF4*) was predicted as a phased locus (Fig. 9d). The fact that the primary cleavage was mediated by ta-siRNA D8(+) suggested that this was not a case of miRNA-mediated phasing process. Therefore, the phased locus observed here may produce secondary siRNAs through the RDR6-DCL4 pathway triggered by D8(+) pha-siRNA.

## Discussion

Next-generation sequencing has tremendously increased the ability for analysis of sRNA. To date, much research has focused on the identification and annotation of miRNAs. However, the majority of expressed sRNAs are not miRNAs. In *Arabidopsis*, miRNAs account for approximately 10 % of the genome. In pear winter buds, only 169 loci (fewer than 0.1 %) were annotated as miRNAs among a total of 218,050 sRNA loci. Because we used ShortStack, which employs a relatively high stringent algorithm, to annotate *MIR* loci, the actual number of miRNA loci may be a bit larger; nonetheless, it was clear that a large number of sRNA loci were not miRNAs. Among these unannotated loci, HP loci represented a small portion. HP loci have long been used as a tool to manipulate plant mRNA expression levels. Unlike the loci that produce miRNAs, sRNAs from HP loci are relatively evenly distributed among the sequences. To date, although a few endogenous HP loci have been characterized in detail, including *Arabidopsis IR71*, *IR2039* [38] and maize *Mu killer* [39], these loci require further studies. As shown in Fig. 2, the majority of sRNA loci in the pear genome cannot form the hairpin structure (i.e. they are non-HP); these non-HP loci include pha-siRNA loci and hc-siRNA loci, among others. Several phased loci, such as miR390-targeted *TAS3* loci and miR482-targeted *NBS-LRR* loci, are conserved among species. This work identified 113 phased loci, among which a conserved *TAS3* locus was identified. Interestingly, ta-siRNA generated from the *TAS3* locus further triggered the production of pha-siRNAs in the upstream region of the target gene *ARF4*. Recently, similar events have also been observed in soybean: transcripts of *ARF3* and *ARF4* were not only cleaved by ta-siARFs 7D(+) and 8D(+), but the ARF targets produced pha-siRNAs [40]. Thus, our work confirmed that siRNAs can function as pha-siRNA triggers, not only in Solanaceae, but also in Rosaceae.

The expression of conserved miRNA differed among tissue and organs. Previous reports identified apple miRNA expression in various tissues [22], comparison with which suggests distinct profile of miRNA in pear winter buds. Although Xia et al. [22] and the present work both used RPM to normalize raw reads for expression level, they used genome-mapped reads while we used total clean reads. It is therefore impossible to directly compare the absolute abundance of each miRNA family. We alternatively compared the five most abundant miRNA families in apple organs and pear winter buds. In pear, miR166 and miR167 were the most abundant sRNAs, followed by miR168, miR319 and miR390. In apple organs, miR166 were relatively highly expressed in leaf and root and miR167 also showed relatively high expression in all tested organs except fruit. However, the miR319 family was not abundantly expressed in apple and miR390 showed similarly low expression, except in flower. Such results suggested that miR319 and miR390 play specific roles in the development of winter buds. miR319 targets class II *TCP* family transcription factors, which function in the coordination of cell proliferation, the differentiation of several morphological traits, the biosynthesis of phytohormons, as well as the regulation of circadian clock rhythms [41]. However, the roles of *TCP* genes in winter buds have not been well described. Compared to other tissue/organs, the high abundance of miR319 suggested relatively low *TCP* expression, which is probably correlated with the active cell division observed in dormant buds, comparing to mature leaves in which cell division is limited to the area near the petiole. In *Arabidopsis*, miR160 and miR167 are involved in auxin signaling via regulation of *ARF* genes [42]. In Japanese pear, we also identified seven and six *ARF* encoding genes regulated by miR160 and miR167, respectively. In addition, a class of ta-siRNAs, produced from the *TAS3* gene and triggered by miR390, were also involved in *ARF* gene regulation via mRNA cleavage. In *Arabidopsis* and wheat, *ARF* maintains seed dormancy by stimulating the ABA signal [43]. Although the functions of auxin and *ARF* on endodormancy have not yet been described in detail, the relatively high expression of *ARF*-target miRNAs, such as miR167 and miR390, suggests their possible roles in the winter bud dormancy.

Many of the previous reports on sRNA uses a single biological sample for differential expression analysis, potentially increasing the risk of false positive results. In our work, we analyzed the sRNAome of samples from two successive seasons as biological replicates, allowing us greater confidence in the results of our differential expression analysis. Differential expression analyses usually employ one of three strategies: i) individual sequences of interest, such as miRNAs; ii) genomic elements, such as genes or transposons; or iii) discrete sRNA-generated genomic loci (bin or cluster) [44]. In this work, we employed two separate strategies: strategy i was applied to miRNA analysis and strategy ii was applied to analyses of other sRNA loci.

During the preparation of our manuscript, Niu et al. [45] reported the analysis of the miRNAs of Chinese white pear during the endodormancy release. They found several miRNAs that were differentially expressed during endodormancy release. Specifically, they identified a pear-specific miRNA miR6390 that directly cleaved the transcript of *PpDAM1* gene, suggesting the post-transcriptional regulation of *PpDAM1*. However, neither the conserved miRNAs nor the pear-specific miRNAs were differentially expressed using samples of two successive seasons in our study. In addition, we did not found the miR6390 in all our four datasets even we sequenced the sRNAome in a relatively high depth. The reason for such differences is probably due to the different cultivars used in these two studies, suggesting that the functions of miRNAs during endodormancy release may vary among pear cultivars.

In the 1540 differentially expressed loci, over 80 % were upregulated after endodormancy release. Considering that a similar phenomenon was observed for the RNA-seq analysis using the same samples, these results might suggest universal transcriptional upregulation following endodormancy release. In total, 20 loci, all of which were upregulated in ecodormancy, were annotated as exons of 19 genes, suggesting their potential regulation of those genes. However, only two genes were identified as differentially expressed in endodormancy vs. ecodormancy. One of them encoded a bZIP protein, while the other was not able to be annotated. The bZIP protein is similar to *Arabidopsis* AtbZIP9, belonging to group C, which has an extended leucine zipper with up to nine heptad repeats. The function of AtbZIP9 has not yet been well characterized, but other members of group C were involved in regulation of seed storage protein production and responses to environmental or pathogen challenges. In the ‘Kosui’ RNA-seq libraries, the *bZIP9* gene was upregulated at ecodormancy, while in the Chinese pear datasets high expression levels were observed in December and January. These results suggest a potential function of *bZIP9* in the regulation of endodormancy release.

There are several cases for which multiple miRNAs accumulate from the same precursor, including cases where miRNA and miRNA* species accumulate to approximately equal levels, cases in which overlapping but distinct miRNA species are produced from the same arm of a stem-loop, and cases where multiple miRNA/miRNA* duplexes are sequentially excised. In this work, we identified a novel miR168 miRNA sequence which overlapped with the well-described miR168. In accordance with the nomenclature proposed in Meyers et al. [46], we designated the original miR168 and novel miR168 in Japanese pear as *Pp*miR168.1 and *Pp*miR168.2, respectively. A similar case was observed for *Arabidopsis* miR161, which targets the *PPR* gene family; the miR161 locus encodes two overlapping miRNAs, miR161.1 and miR161.2, from a contiguous 29 nt sequence. Considering the evolution of this type of *MIR* locus, Allen et al. [47] proposed that the MIR161 gene evolved relatively recently via an inverted duplication event associated with the active expansion of the target gene. The similar structure of pear miR168 suggests the same mechanisms of accumulation and evolution as those of *Arabidopsis* miR161. In pear, miR168.1 was the dominant mature miRNA, accounting for over 70 % of sRNA, while miR168.2 accounted for only ~5 %. However, PARE-seq showed that most cleavage events in the target gene *AGO1* were mediated by miR168.2 but not by miR168.1, possibly due to differences in minimum free energy required for forming a duplex between the miRNA and the target mRNA.

As mentioned above, miR390 triggered the production of ta-siRNAs, which post-transcriptionally regulate *ARF* genes. Previous reports show reveal at least five *TAS3* loci in the apple genome [22]. Despite pear belonging to the same genus as apple, our phased loci analysis found only one *TAS3* locus sharing high homology with apple *TAS3-1* genes. Further Blasting with core D7(+) and D8(+) sequences to pear genome sequences also matched only a single locus (data not shown). We do not consider only one *TAS3* locus to be present in pear genome and attribute our result to the incomplete pear genome used in this study. In addition, the lack of pear EST information also limited us to identified *TAS* genes and other non-coding RNAs. Compared to apple, for which 339,058 EST fragments exist in the Genbank EST database, only 4413 EST fragments exist for pear (as of December, 2014), none of which correspond to the conserved *TAS* locus. In contrast, four of five apple *TAS* loci were identified from the EST database [22].

Much work has reported identification of miRNAs in new plant species. In this study, we used three pipelines (miRProf and miRCAT, which are parts of UEA sRNA workbench, and ShortStack) to annotate miRNAs. miRProf uses similarity to search for matches while ShortStack and miRCAT identify miRNA sequences *de novo*. Utilizing annotations of known miRNAs, miRProf identified 137 miRNAs belonging to 32 known miRNA families, while ShortStack identified only 68 known miRNAs belonging to 22 families. Although BLAST-based strategies are more sensitive for identification of known miRNAs, they are unable to obtain information on miRNA location and copy number in the genome and their sensitivity depends largely on the BLAST parameters chosen (with or without mismatches), especially for species with no information in miRBase. In contrast, the *de novo* strategies use a series of criteria to determine the probability of each sRNA and their accuracy depends on the stringency of the criteria used. In recent years, the criteria for miRNA annotation have largely been changed. For example, most miRNA precursors produce more than a single product (as in the example of miR168), which are not able to be properly annotated using all three pipelines. Therefore, it is necessary to develop a more robust algorithm for plant miRNA annotation.

## Conclusions

We identified 137 conserved or less conserved miRNAs and 50 pear-specific miRNAs. However, none of the conserved microRNAs or pear-specific miRNAs was differentially expressed between endodormancy and ecodormancy stages at least in this study. On the contrast, 1540 of 218,050 loci that produced sRNAs were differentially expressed between endodormancy and ecodormancy. We also characterized a multifunctional miRNA precursor *MIR168* and we showed that siRNAs are able to trigger phased siRNAs in pear like reported in other plant species. Our work showed that dormancy release is a highly coordinated physiological process involving the regulation of sRNAs.

## Methods

### Plant materials

All plant materials used in this study were described in Bai et al. [27]. The total RNA used for transcriptome analysis in Bai et al. [27] was also used for sRNA-seq in this work. For PARE-seq, buds sampled on Dec. 6 (under endodormancy) and on Dec. 31 (after endodormancy release) in the 2009/2010 seasons and on Dec. 2 (under endodormancy) and on Dec. 31 (after endodormancy release) in the 2010/2011 season were mixed in equal quantities and used for RNA preparation.

### RNA extraction and deep sequencing

Total RNA for sRNA-seq was prepared as described in Bai et al. [27]. Deep sequencing of the degradome was carried out following the method described in German et al. [48] by BGI (Shenzhen, China).

### Bioinformatics analysis of small RNAs

All the raw reads were first processed to removing the 3′ adaptors with the free script cutadapt (https://cutadapt.readthedocs.org/en/stable/). Any sequences without the adaptor matched were excluded from further analysis. The reads were then filtered to remove the reads <18 or >25 nt, with low complexity or matching to tRNAs and rRNAs by the filter function integrated in the UEA sRNA workbench [34]. The resulting clean reads were subjected to the analysis using two well-established pipelines, UEA sRNA workbench and ShortStack. For the UEA sRNA workbench, conserved miRNA analysis was performed with miRProf with no mismatch allowed using miRbase v21 [35]. The conserved miRNAs were then removed from the libraries and the remaining sRNAs were subjected to the pear-specific miRNAs identification using miRCat using the default plant parameters. The pear genome sequences were retrieved from the Pear Genome Project [30]. The total number of the reads in a given library was used for the normalization of read abundance, denoted as reads per million reads (RPM). In addition, the clean reads were also mapped to pear genome sequences with bowtie (v1.0.0, [31]) and subjected to the pipeline ShortStack (v1.2.4, [32]) with the default parameters to *de novo* identify the miRNAs loci, HP sRNA loci and other sRNA loci. The miRNA loci were further annotated with miRBase to identify the conserved miRNAs and pear-specific miRNAs. To identify the phased siRNA loci, the p-value (Phase pval) calculated by ShortStack were adjusted to false discovery rate (FDR) using Benjamini-Hochberg method and the sRNA loci with the FDR < 0.05 were determined as phased loci. The raw counts for each sRNA loci calculated by ShortStack were then used for differential expression analysis.

### Differential expression analysis of miRNAs and sRNA loci

Differential expression of conserved, less conserved and pear-specific miRNAs were carried out using edgeR [36]. The raw counts of all individual sRNA tags, including all types of sRNAs, were calculated and used as the input of edgeR. The edgeR output was then manually adjusted to summarize the counts of miRNAs within the same family. Differential expression of the sRNA loci were calculated by edgeR using the output of ShortStack as the input.

### Bioinformatic analysis of PARE data

Mixed RNA with equal amounts of the four samples used for sRNA-seq was used for PARE sequencing. After adapter-trimming and genomic mapping as done for the sRNA data. The pipeline PAREsnip [33] within the UEA sRNA workbench was used for the PARE analysis. The threshold for the alignment score was set to 4.5 for all miRNAs. Only category 0–3 were analysed to minimize the false positive results. The pear gene set was retrieved from Pear Genome Project and the annotation of the genes was carried out by BLASTx alignment to *Arabidopsis* genes (TAIR10). GO analysis was carried out with BiNGO [49], a plugin of Cytoscape.

### qRT-PCR of miRNAs

Total RNAs were extracted using the method previously described [27]. cDNAs synthesis and qRT-PCR were performed with Mir-X miRNA qRT-PCR SYBR kit (Takara, Tokyo, Japan) according to the manufacturer’s instruction. Relative expression was determined with the 2^-ΔΔT^ algorithm by normalizing to the plant U6 non-coding RNAs. The miRNA-specific primers used for real-time RT-PCR are the sequences of each mature miRNAs.

## Availability of supporting data

All the raw sequencing data used in this work have been submitted to the NIH Short Read Archive (SRA) under accession number PRJNA310384.

## Additional files

Additional file 1: Table S1. Information of sRNA clusers annotated by ShortStack. (XLSX 28366 kb) Additional file 2: Table S2. PAREsnip identified the target of small RNAs. (XLSX 672 kb) Additional file 3: Table S3. Target of known miRNAs in pear identified by PARE-seq. (XLSX 38 kb) Additional file 4: Figure S1. Step-loop structures of pear-specific miRNAs predicted by RNAfold. (PDF 4170 kb) Additional file 5: Table S4. Abundance of novel miRNAs in each libary. (XLSX 57 kb)
