# Supplementary material for: Small RNA and PARE sequencing in flower bud reveal the involvement of sRNAs in endodormancy release of Japanese pear (Pyrus pyrifolia 'Kosui')
Source: BMC Genomics. 2016 Mar 15;17:230. doi: 10.1186/s12864-016-2514-8 (PMC4791883; doi:10.1186/s12864-016-2514-8)
Supplement: Additional file 4: Figure S1. — Step-loop structures of pear-specific miRNAs predicted by RNAfold. (PDF 4170 kb) [file 12864_2016_2514_MOESM4_ESM.pdf]

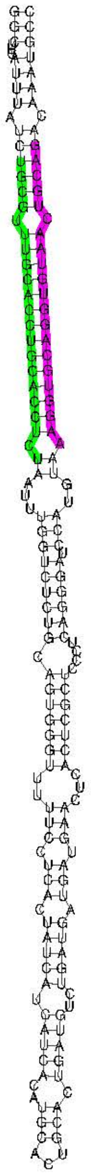

TGCGTTTGCACCTGCACCTCT_scaffold309.0_28164-28184

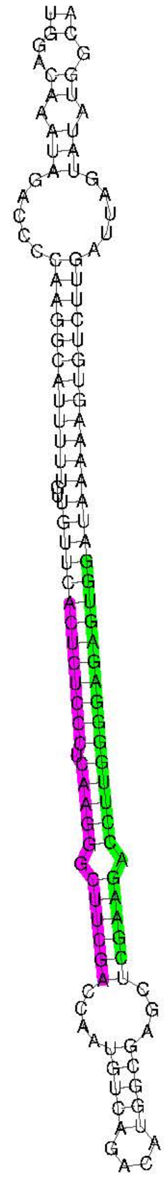

CGAAGACCTTGGGGAGAGTGG_scaffold21.0_451983-452003

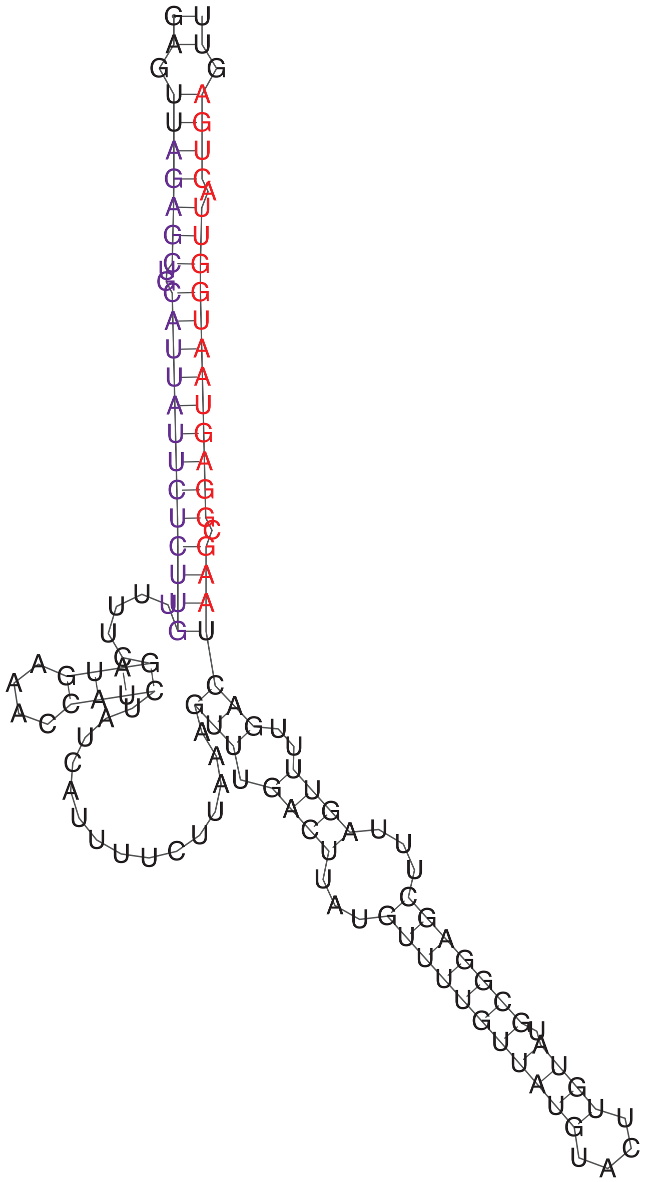

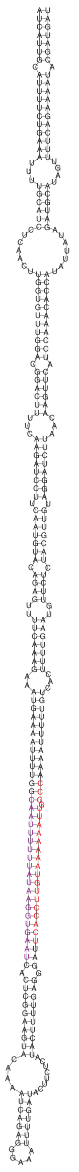

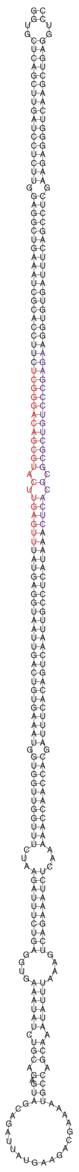

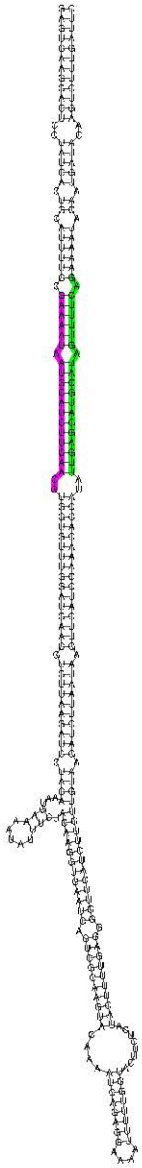

TTGAGGATGCATAGTTTTCAG_scaffold266.0_383741-383761

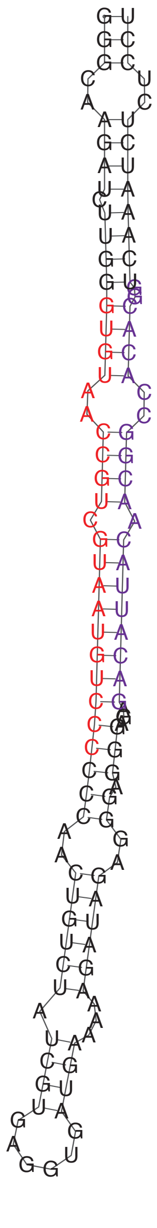

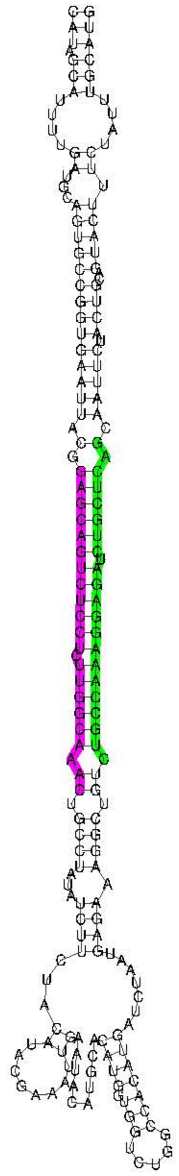

CTGCCAAAGGAGATCTGCTCAG_scaffold1.0_3618550-3618571

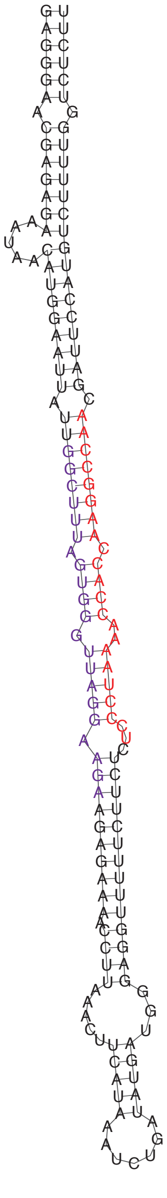

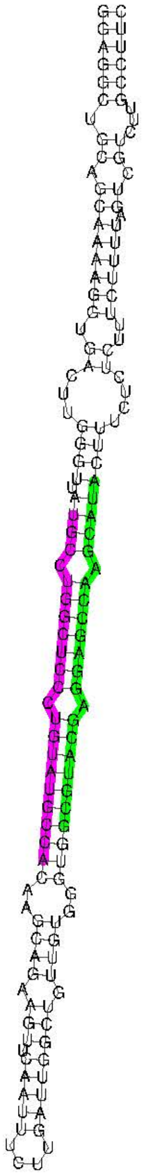

GCGTACGAGGAGCCAAGCATA_scaffold32.0_439587-439607

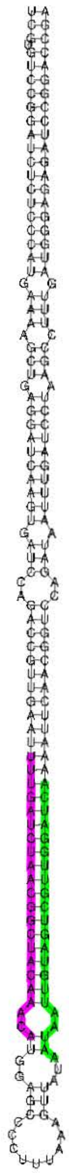

AATAATTGTAGTCGTTGGATCA_scaffold371.0_364491-364512

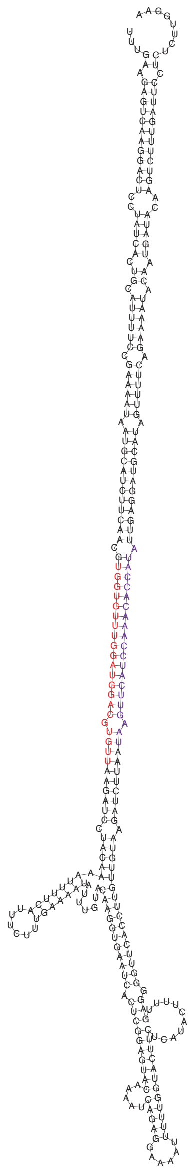

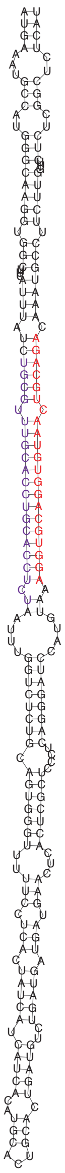

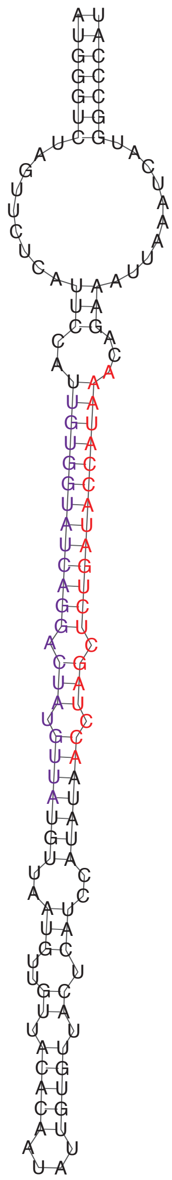

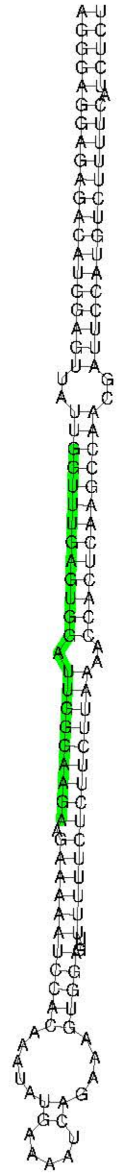

GGTTTGAGTGGATTGGGAAGA_scaffold2.0_3517165-3517185

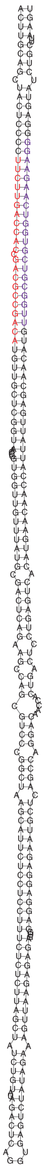

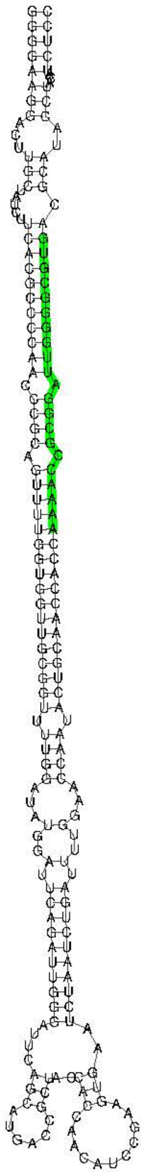

AAAACCGCGGATTGGGGCGTG_scaffold17.0_1106089-1106109

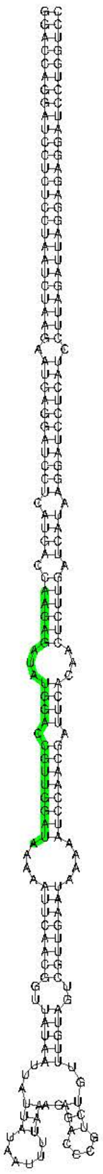

AAGAGATATGGACCGTTGGATA_scaffold1.0_3711548-3711569

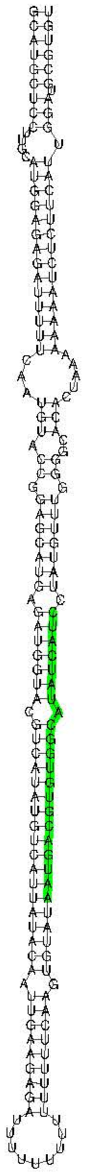

AATGACGTGTGGCATATCATC_scaffold28.0_546699-546719

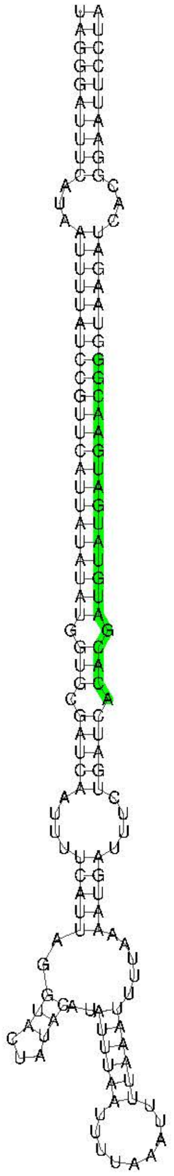

ACACGATGTATGATGAACGG_scaffold1044.0_101372-101391

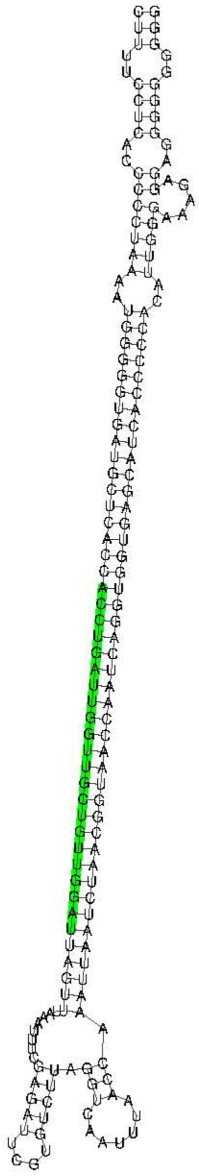

ACCTGATTGGTTGCTGTTGGAT_scaffold89.0_380874-380895

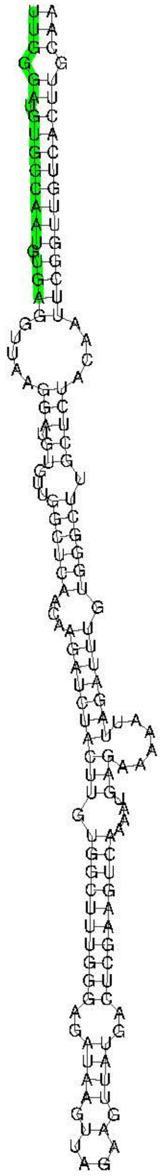

ACTTTGGGATGTGGCAATGTGA_scaffold285.0_183708-183729

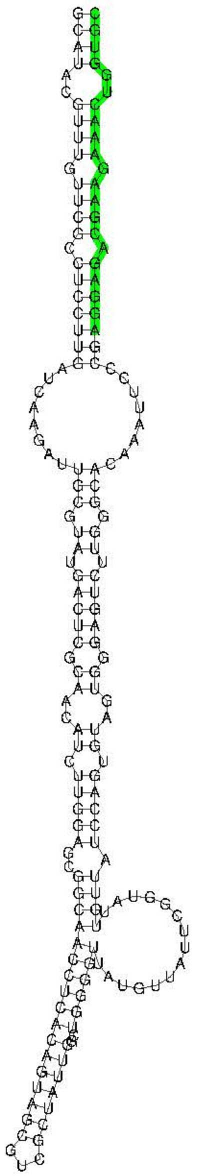

AGGAGACGAAGAAACTGGTGC_scaffold463.0_226452-226472

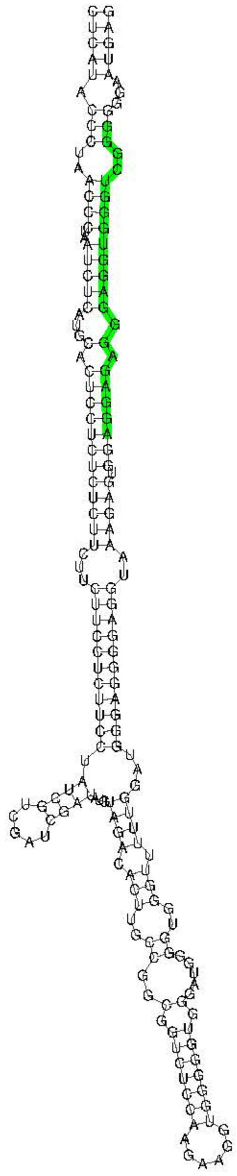

AGGAGAGGGAGGTGGGTCGGG_scaffold411.0_139391-139411

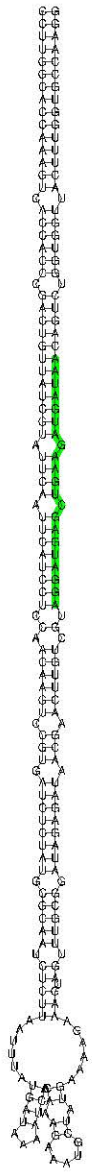

AGGATGAGCTGAAGATGATAA_scaffold1.0_2288859-2288879

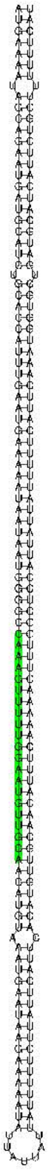

CAAAGTTTTTGGAATGTTGCA_scaffold54.2_447463-447483

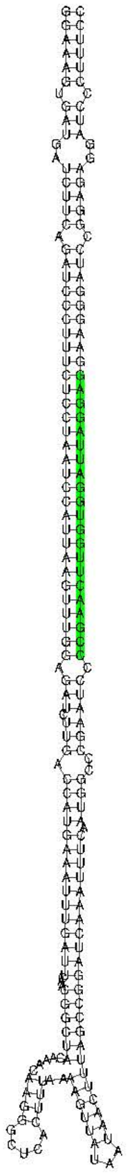

CCGAACTTGGTGGATTAGGAG_scaffold1042.0_77121-77141

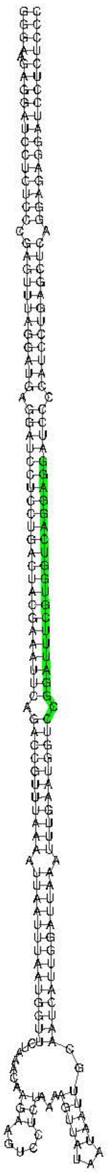

CCGGATTTCGTGGTCAGGAGG_scaffold124.0_491254-491274

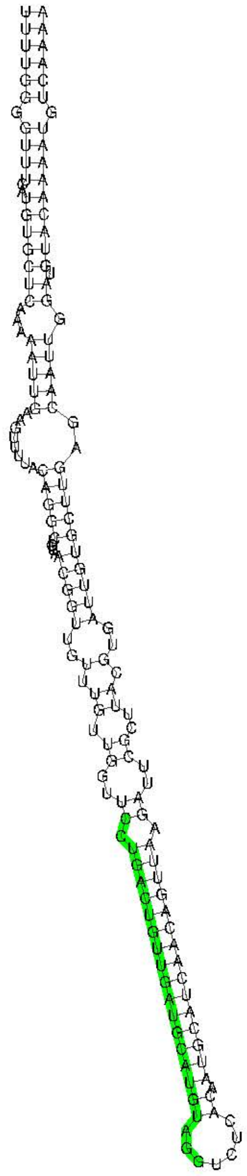

CCTGACTGTTGATGCATGTAGG_scaffold373.0_139290-139311

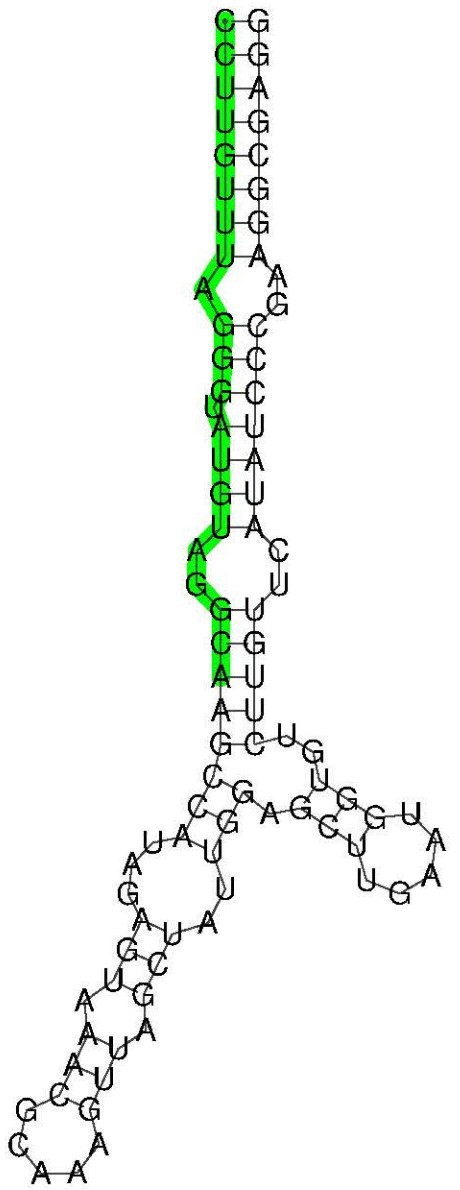

CCTTGTTTAGGGTATGTAGGCA_scaffold1113.0_55073-55094

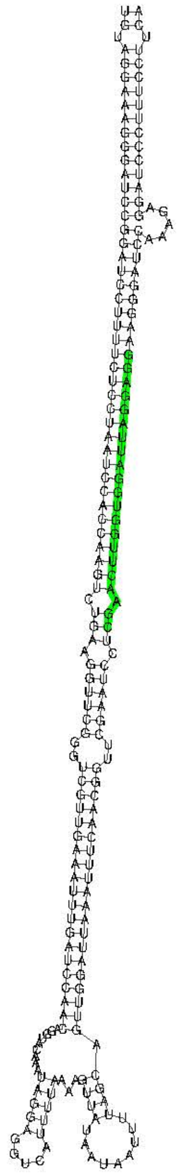

CGAACTTGGTGGATTAGGAGG_scaffold39.0_1241288-1241308

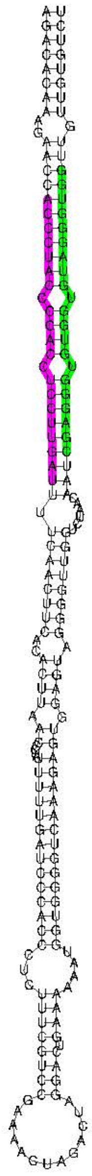

CGAGGGTGTGGTGTAGGGTGG_scaffold622.0_272702-272722

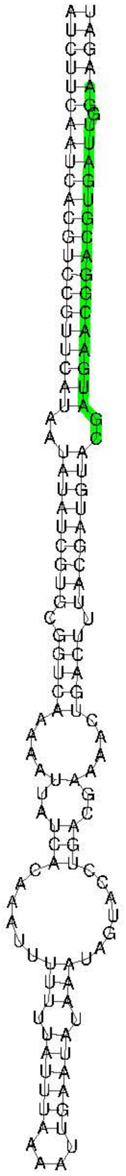

CGATGAACGGACGTGATTGGA_scaffold206.0_482311-482331

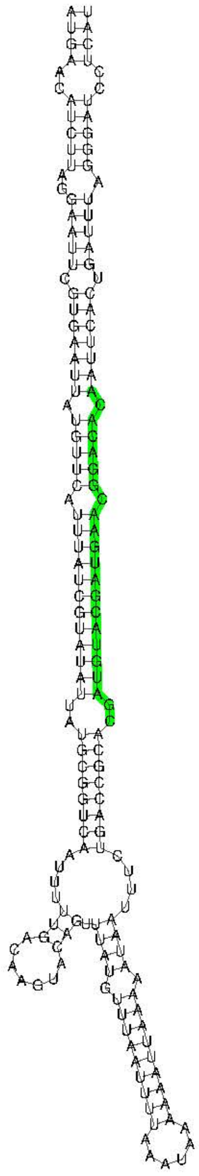

CGATGTACGATGAACGGACACA_scaffold1279.0_75464-75485

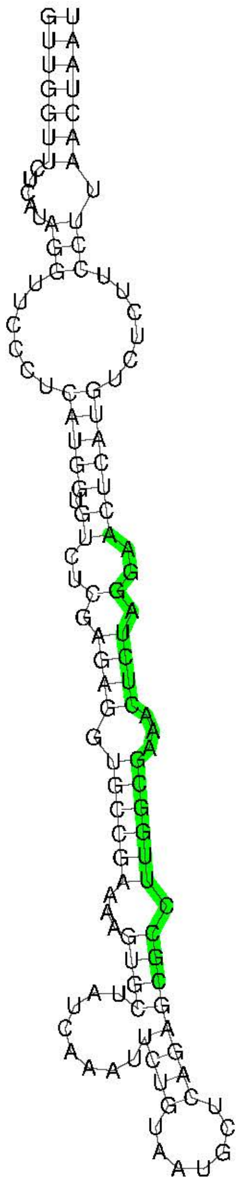

CGCCTTGGCGAAACTCTAGGAA_scaffold310.0_259627-259648

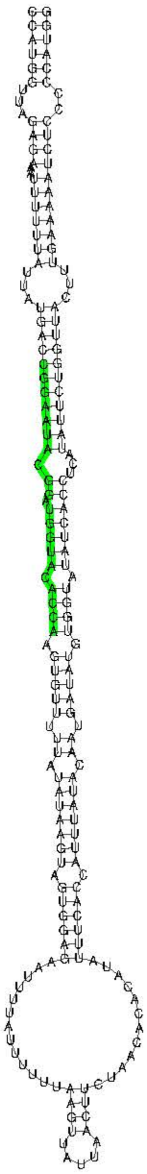

CGGAATACGGATGGTACACCA_scaffold63.0_950578-950598

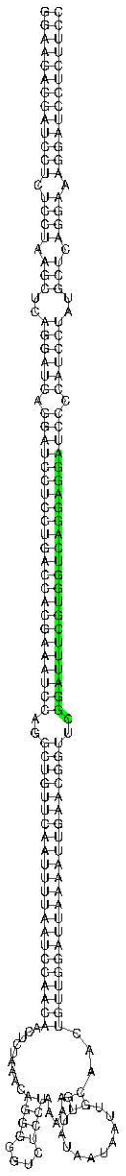

CGGATTTCGTGGTCAGGAGGA_scaffold689.0_117843-117863

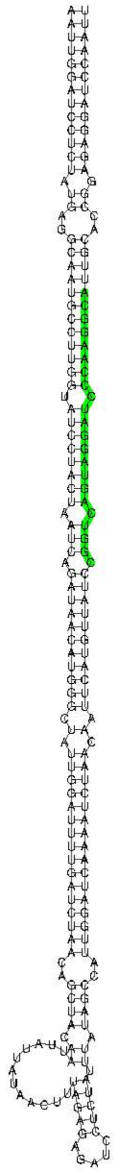

CGGTCAGTAGGATCCCAAGGCA_scaffold242.0_185346-185367

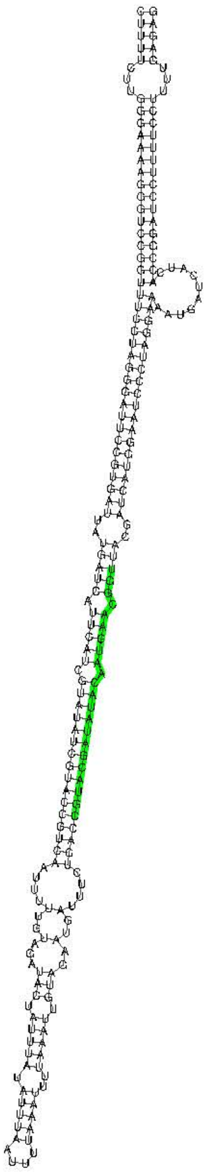

CGTACGATATACAATGAACGGT_scaffold171.0_72510-72531

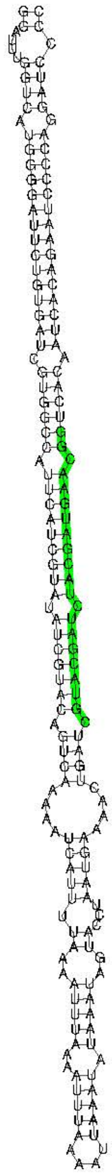

CGTACGATCTACGATGAACGG_scaffold182.0_668377-668397

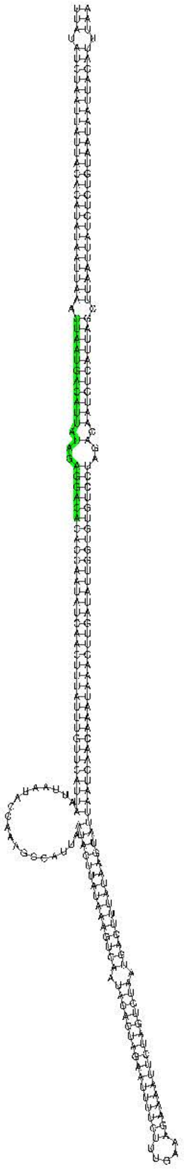

CTAATGACATTATAGAGGACA_scaffold103.0_807727-807747

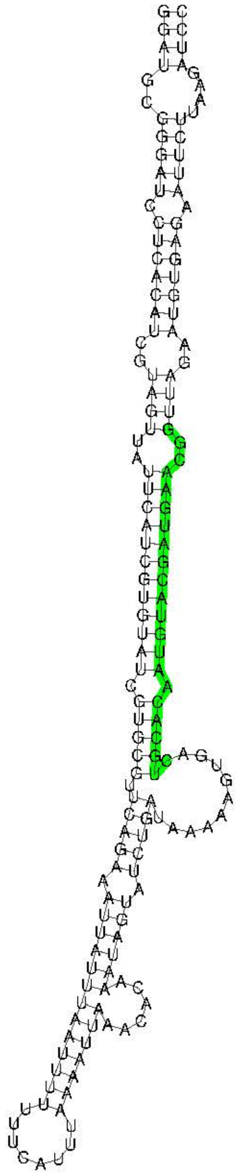

CTGCACAATGTACGATGAACGG_scaffold13.0_1394637-1394658

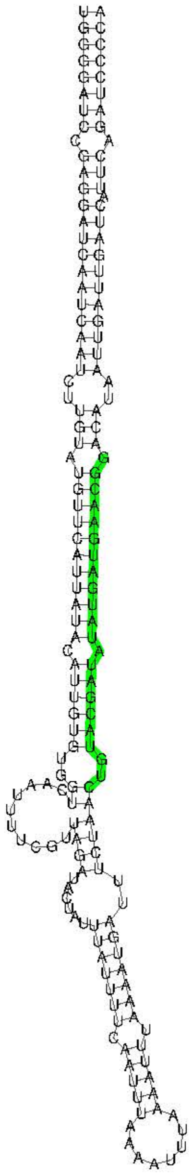

CTGTACGATATATGATGAACGG_scaffold1196.0_89625-89646

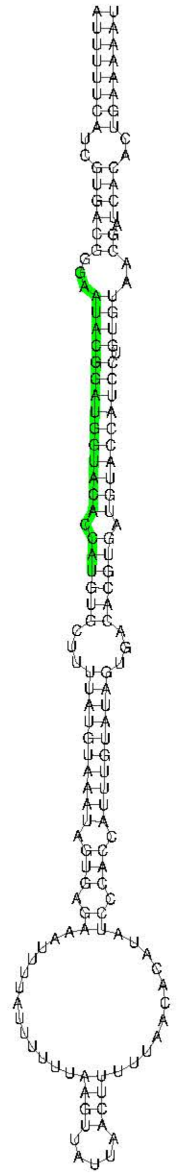

GAATACGGATGGTACACCAT_scaffold330.0_248865-248884

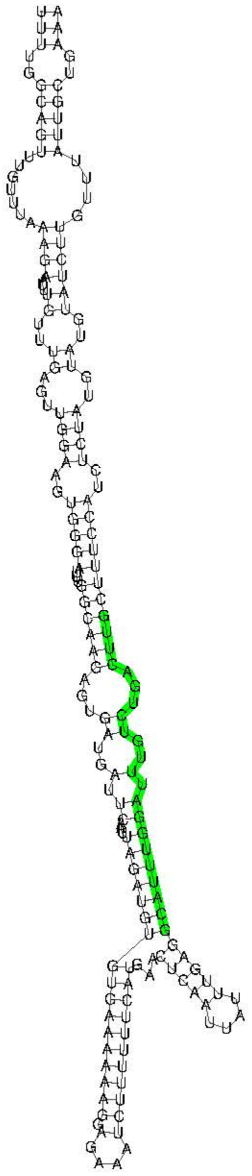

GCATTTGGATTTGTCTGACTTG_scaffold1206.0_10164-10185

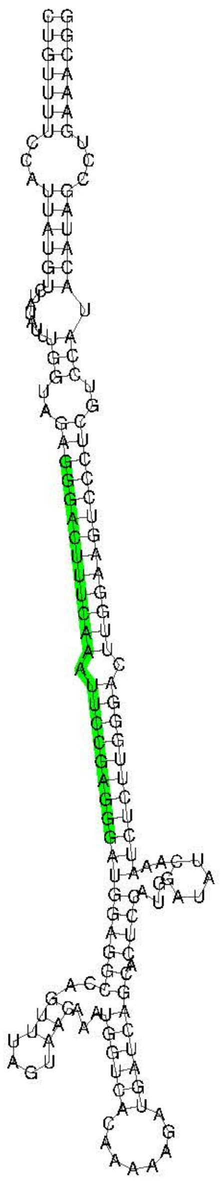

GGGACTTTCAAATTCCGAGGG_scaffold353.0_423235-423255

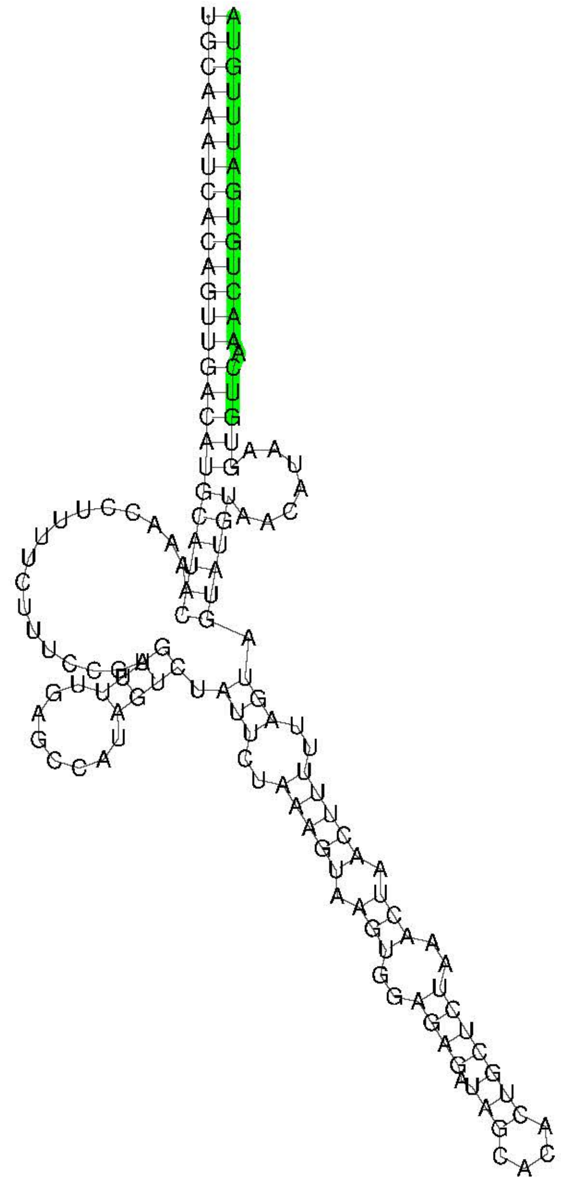

GTCAAACTGTGATTTGTAGGCA_scaffold94.0_553323-553344

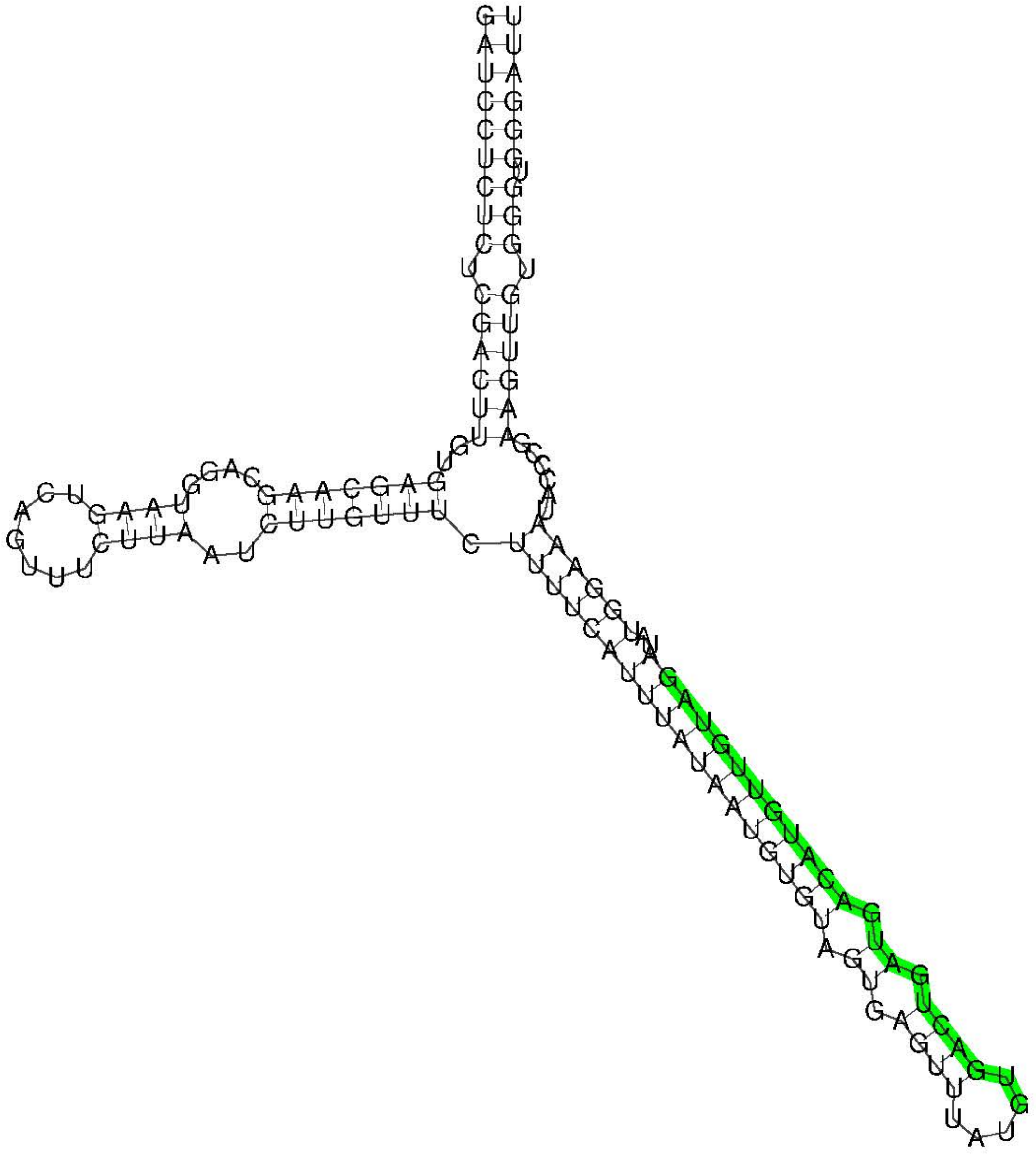

GTGACTGATGACATGTTGTAG_scaffold322.0_224220-224240

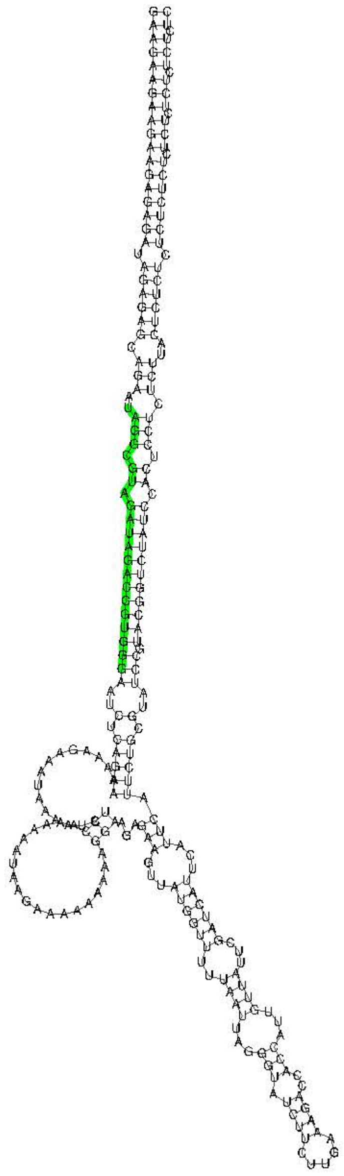

TAGGCGTAGATAGACCGTGGG_scaffold144.0_39353-39373

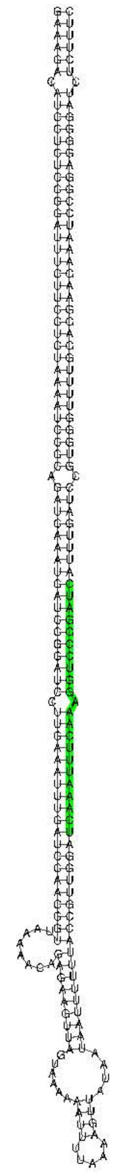

TCAAATTTCAAAGGTCCGGATC_scaffold220.0_399722-399743

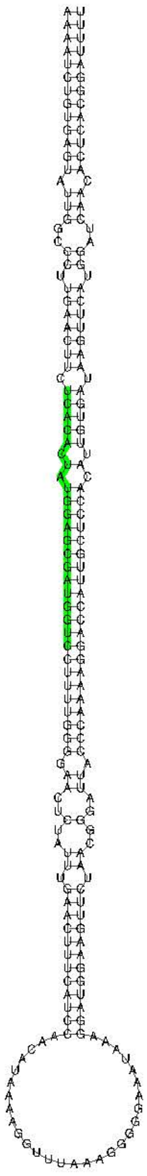

TCACACTATGGAGCGATGGTC_scaffold1.0_57679-57699

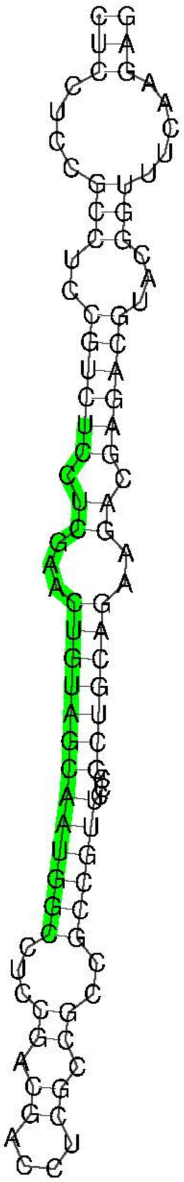

TCCTCGAACTGTAGCAATGGC_scaffold44.1_36177-36197

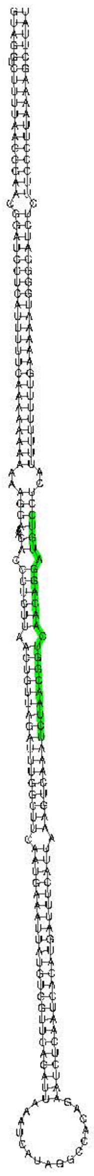

TCTAACGGTCAAGAGGATGTC_scaffold751.0_24240-24260

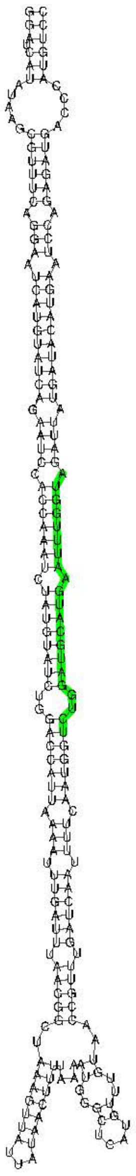

TCTGGATGCATGAATTTGGTA_scaffold83.0_405508-405528

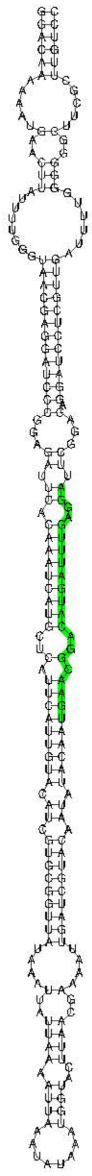

TGAACGGACATGATTTGAGGA_scaffold3.0_371612-371632

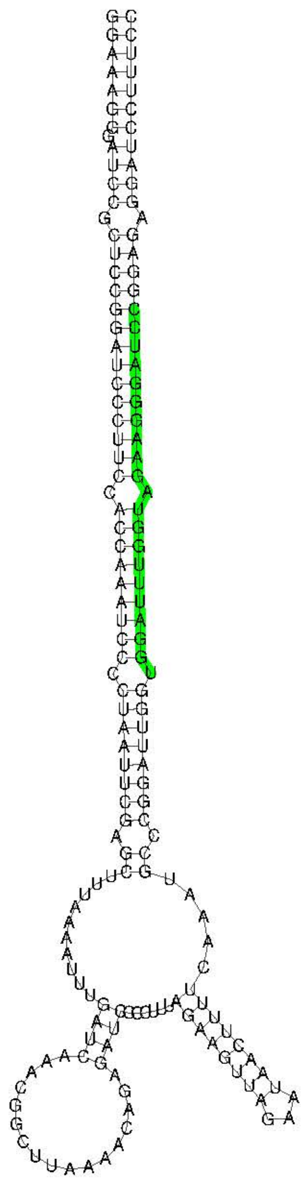

TGGATTTGGTAGAAGGGATCC_scaffold1279.0_53473-53493

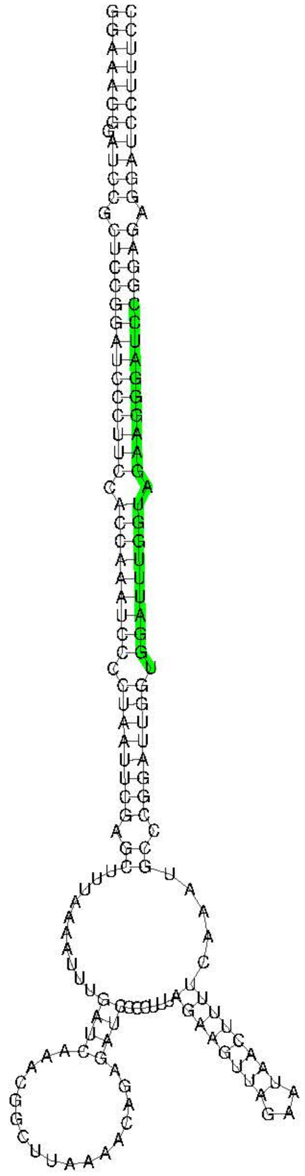

TGGATTTGGTAGAAGGGATCC_scaffold1279.0_53473-53493

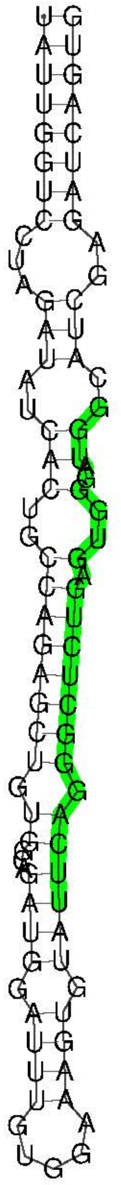

TTCAGGGCTCTGAGTGGGATGG_scaffold48.0_391355-391376

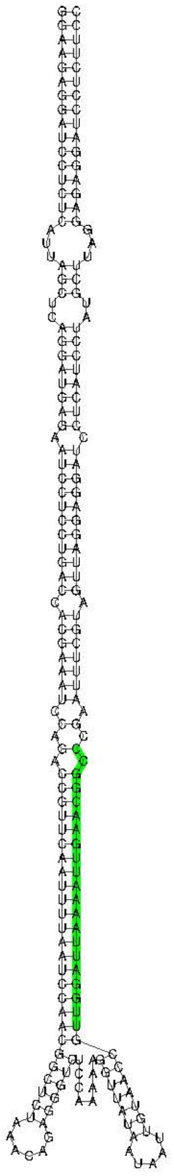

TTGGATTAAAATTGAACGGCC_scaffold1.0_124875-124895
